# Fundamental Equation of State for Fluid Tetrahydrofuran

**DOI:** 10.1007/s10765-023-03258-3

**Published:** 2023-10-09

**Authors:** Felix Fiedler, Joel Karog, Eric W. Lemmon, Monika Thol

**Affiliations:** 1 Lehrstuhl für Thermodynamik, Ruhr-Universität Bochum, Universitätsstraße 150, 44801 Bochum, Germany; 2 Applied Chemicals and Materials Division, National Institute of Standards and Technology, 325 Broadway, Boulder, CO 80305 USA

**Keywords:** Equation of state, Helmholtz energy, Tetrahydrofuran, Thermodynamic properties, THF

## Abstract

An empirical fundamental equation of state in terms of the Helmholtz energy for tetrahydrofuran is presented. In the validity range from the triple-point temperature up to 550 K and pressures up to 600 MPa, the equation of state enables the calculation of all thermodynamic properties in the liquid, vapor, and super-critical regions including saturation states. Based on an extensive literature review, experimental data are represented within their experimental uncertainty. In the homogeneous liquid phase at atmospheric pressure, the uncertainty in density is 0.015 %, speed of sound is represented with an uncertainty of 0.03 %, and isobaric heat capacity has an uncertainty of 0.4 %. Isobaric heat capacities in the homogeneous vapor phase are described with an uncertainty of 0.2 %. Higher uncertainties occur above atmospheric pressure for all homogeneous properties. Depending on the temperature range, vapor pressure can be calculated with an uncertainty from 0.02 % to 3 %. The extrapolation behavior is evaluated, showing reasonable extrapolation behavior towards extreme conditions.

**Supplementary Information:**

The online version contains supplementary material available at 10.1007/s10765-023-03258-3.

## Introduction

**Fig. 1:**
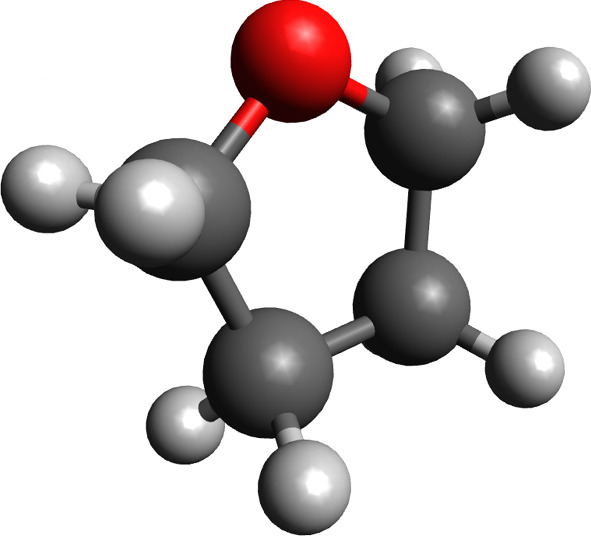
Molecular structure of tetrahydrofuran [4]. Atoms are colored in red (oxygen), dark grey (carbon), and light grey (hydrogen)

Tetrahydrofuran (THF, ${\text{C}}_4{\text{H}}_8{\text{O}}$) is a five-node cyclic ether that is internationally listed with the CAS registry number 109-99-9, and is also known by several synonyms like oxolane, 1,4-epoxybutane, diethylene oxide, or hydrofuran [1–3]. The molecular structure is displayed in Fig. 1.

THF is used in the chemical industry as a solvent, reaction medium, or starting material for syntheses [3, 5]. One prominent application is the polymerization with simultaneous ring opening to poly(tetramethylene oxide), which is important for the production of elastic construction materials, thermoplastics, and elastomers [1].

Due to its polarity, THF is entirely miscible in water. Its large molecule size favors THF as a promoter for gas hydrates, which means that the conditions of hydrate formation can be decreased to lower pressure regions [6, 7]. Thus, THF can play a key role in the realization of cheap and efficient gas storage for important industry gases, like CO_2_ or H_2_, at applicable pressure conditions [8–10].

In order to develop, validate, or analyze processes in terms of practicability and economic feasibility, the accurate knowledge of thermodynamic properties of the applied fluids is necessary. Nowadays, equations of state (EOS) provide the foundation to calculate thermodynamic properties, e.g., vapor pressure, density, or heat capacities. With major contributions of Span and colleagues, the development of empirical fundamental EOS is now standard in literature. Span [11] distinguishes between accurate reference EOS for well-investigated fluids [12–14], e.g. CO_2_ [15], and technical EOS that meet engineering requirements for technical applications even if the modeled fluids have a poor experimental data base [16–19].

In this work, we present the first empirical fundamental EOS for THF in terms of the reduced Helmholtz energy with temperature and density as independent variables. Being a fundamental property, the expression in terms of the Helmholtz energy enables the calculation of all thermodynamic properties over the entire fluid range including saturation states. Thus, both thermal and caloric properties were used for the development of the present EOS for THF. Most of the available data from the literature are limited to fairly low temperatures at atmospheric pressure. According to Span [11], the present EOS for THF is categorized as a technical EOS. Therefore, during the regression process, emphasis was placed on physically correct extrapolation behavior of the EOS. In most applications, THF appears in fluid mixtures together with other components. The performance of mixture models including THF benefits from well-behaving EOS beyond state regions that are evaluable through experimental data.

## Equation of State

The fundamental equation of state for THF is expressed in terms of the Helmholtz energy. Combining derivatives of the Helmholtz energy according to the independent variables yields a consistent representation of thermal and caloric properties [11]. The use of the Helmholtz energy for the correlation provides a continuous description of thermodynamic properties over the entire fluid phase including for vapor-liquid equilibrium.

The Helmholtz energy *a* is split into an ideal part $a^\circ$ and a residual part $a^\text {r}$

1 $$\begin{aligned} a(T,\rho )=a^\circ (T,\rho )+a^\text {r}(T,\rho ). \end{aligned}$$

Temperature *T* and density $\rho$ are reduced by the critical parameters, and the Helmholtz energy is reduced by temperature and the molar gas constant *R* to obtain a dimensionless equation according to

2 $$\begin{aligned} \alpha (\tau ,\delta )=\frac{a(T,\rho )}{RT}=\frac{a^\circ (T,\rho )+a^\text {r}(T,\rho )}{RT} =\alpha ^\circ (\tau ,\delta )+\alpha ^\text {r}(\tau ,\delta ), \end{aligned}$$

where $\alpha$ is the reduced Helmholtz energy, $\tau$ is the reciprocal reduced temperature $\tau =T_\text {c}/T$, and $\delta$ is the reduced density $\delta =\rho /\rho _\text {c}$. Some fluid-specific parameters and physical constants are given in Table 1.

**Table 1:** Constants and fluid-specific thermodynamic properties of THF

Symbol	Quantity	Value	Unit	Reference
*R*	Molar gas constant	8.314 462 618	$\text {J}\cdot \text {mol}^{\text {-1}}{\cdot }\text {K}^{\text {-1}}$	[20]
*M*	Molar mass	72.1057	$\text {g}{\cdot }\text {mol}^{\text {-1}}$	[2]
$T_{\text {c}}$	Critical temperature	540.2	K	[21]
$p_{\text {c}}$	Critical pressure	5.3045	MPa	This work
$\rho _{\text {c}}$	Critical density	4.4	$\text {mol}{\cdot }\text {dm}^{\text {-3}}$	This work
$T_{\text {nbp}}$	Normal-boiling-point temperature	339.075	K	This work
$T_{\text {tr}}$	Triple-point temperature	164.76	K	[22]
$p_{\text {tr}}$	Triple-point pressure	0.15	Pa	This work
$\omega$	Acentric factor	0.234	–	This work

### Description of the Ideal Gas

The ideal part describes ideal-gas behavior. The Helmholtz energy of the ideal gas can be formulated as

3 $$\begin{aligned} a^\circ (T,\rho )=u^\circ (T)-Ts^\circ (T,\rho ). \end{aligned}$$

The ideal-gas internal energy $u^\circ$ and the ideal-gas entropy $s^\circ$ can be expressed as

4 $$\begin{aligned} u^\circ= & {} u^\circ _0+\int _{T_0}^{T}c^\circ _v\text {d}T \ {\text{and}} \end{aligned}$$

5 $$\begin{aligned} s^\circ= & {} s^\circ _0+\int _{T_0}^{T}\frac{c_v^\circ }{T}\text {d}T-R\ln \left( \frac{\rho }{\rho _0}\right) , \end{aligned}$$

where $c^\circ _v$ is the isochoric ideal-gas heat capacity, $s^\circ _0$ is the ideal-gas entropy at the reference state, and $\rho _0$ is the ideal-gas density at the reference state. For THF, the normal boiling point is chosen as the reference state, which is defined as the saturation temperature $T_0$ and saturated liquid density $\rho _0$ at atmospheric pressure $p_0$.

Combining these two equations into Eq. 3, the ideal-gas Helmholtz energy yields

6 $$\begin{aligned} a^\circ (T,\rho )=u^\circ _0 -Ts^\circ _0 -RT\ln \left( \frac{\rho }{\rho _0}\right) + \int _{T_0}^{T}c^\circ _v\text {d}T-T\int _{T_0}^{T}\frac{c_v^\circ }{T}\text {d}T, \end{aligned}$$

where $T_0$, $\rho _0$, $u^\circ _0$ and $s^\circ _0$ describe the reference state.

According to Eq. 6, a correlation for the ideal-gas heat capacity is needed to calculate ideal-gas properties. Such correlations are usually formulated for the isobaric ideal-gas heat capacity with the form:

7 $$\begin{aligned} \frac{c_p^\circ }{R}=c_0+\sum _{k=1}^{4}m_k\left( \frac{\theta _k}{T}\right) ^2\frac{{\text {exp}}(\theta _k/T)}{\left[ {\text {exp}}(\theta _k/T)-1\right] ^2}. \end{aligned}$$

This formulation presupposes an empirical approach since only the constant $c_0$ is directly connected to physical background. $m_k$ and $\theta _k$ are adjustable parameters. At low temperatures, the contribution of vibrational degrees of freedom to the internal energy is negligible, and, thus, molecules in this state can only store energy in the form of translation and rotation. A molecule like THF, which is a non-linear molecule, can store energy through motion in three translational and three rotational degrees of freedom for temperatures approaching the absolute minimum, where each degree of freedom contributes 1/2 *R*. This results in the isochoric ideal-gas heat capacity $c_v^\circ =6/2$ *R* at low temperatures. According to Eq. 7, only the constant $c_0$ accounts for this temperature-independent contribution. Transforming the isochoric heat capacity to the isobaric heat capacity yields: $c_0=(c_v^\circ +R)/R=8/2=4$. [11]

With increasing temperatures, the so-called Planck–Einstein terms describe the temperature dependency of the ideal gas. Although physically based, the parameters $m_k$ and $\theta _k$ are fitted to $c_p^\circ$ data points that are often simulated or derived from experimental measurements. Their values are listed in Table 2.

8 $$\begin{aligned} \alpha ^\circ (\tau ,\delta ) = c^{\text{I}} + c^{\text{II}}\tau + \ln \delta + (c_0-1)\ln \tau + \sum _{k=1}^{4} m_k\ln \left[ 1-\exp (-\theta _k\tau /T_{\text {c}})\right] \end{aligned}$$

**Table 2:** Parameters of the ideal part of the EOS for THF, c.f. Equations 7 and 8

*k*	$m_k$/-	$\theta _k$ (K)
1	18.2	1460
2	11.394	3461
3	1.05	11 000
4	2.37	517
$c^{\text{I}}$	2.91 973 647 056 971	
$c^{\text{II}}$	− 1.38 409 803 793 207	

Equation 8 combines Eqs. 6 and 7 with the relation $c_v^\circ = c_p^\circ -R$. The integration constants $c^{\text{I}}$ and $c^{\text{II}}$ ensure the reference point conditions are met.

**Fig. 2:**
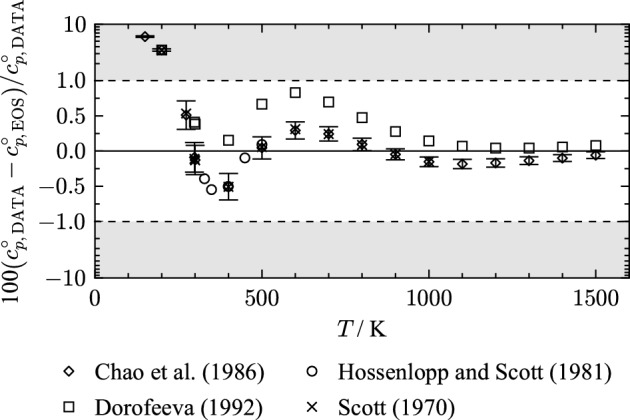
Percentage deviations of ideal-gas isobaric heat capacities from values calculated with the present EOS as a function of temperature. The ordinate is linearly scaled between the dashed lines and logarithmically scaled in the gray filled regions

For the determination of $\alpha ^\circ$, Eq. 7 is adjusted to $c_p^\circ$ literature data. The underlying data base for ideal-gas heat capacities is shown in Fig. 2. The data of Chao et al. [23], Hossenlopp and Scott [24], and Scott [25] are very consistent, while the data of Dorofeeva [26] exhibit a systematic offset from the others.

Hossenlopp and Scott [24] use $c_p$ data with an assigned expanded uncertainty of 0.4 % ($k= 2$) to derive ideal-gas heat capacities $c_p^\circ$. The AARD of the $c_p^\circ$ data set is 0.33 % with a maximum deviation of 0.55 % at 350 K. The other publications used statistical thermodynamics to determine $c_p^\circ$. Chao et al. [23] provide uncertainties for each data point that originate from applying different methods for the calculation of pseudo rotational energy levels. Figure 2 shows that the data of Chao et al. [23] are not represented within their uncertainties. This is because focus was placed on the accurate representation of the real-gas isobaric heat capacities of Hossenlopp and Scott [24] in the gaseous phase (see Sect. 3.6). In the gaseous phase at low pressures, the isobaric heat capacity and the ideal-gas isobaric heat capacity are nearly identical. The alignment with $c_p$ data of Hossenlopp and Scott [24] implies different values of $c_p^\circ$ than reported by Hossenlopp and Scott [24], Chao et al. [23], and Scott [25] in the temperature region from 328 K to 500 K.

In the temperature region above 500 K, the ideal-gas isobaric heat capacity description is in good agreement with the data of Chao et al. [23].

### Description of the Real Fluid

**Table 3:** Parameters of the residual part of the EOS for THF, cf. Equation 9

*i*	$n_i$	$t_i$	$d_i$	$l_i$	$\eta _i$	$\beta _i$	$\gamma _i$	$\epsilon _i$
1	0.04 386	1	4					
2	0.766	0.12	1					
3	− 1.2 355 036 286 776	0.94	1					
4	− 0.6 899 995 453 364	1.111	2					
5	0.201 742	0.41	3					
6	− 0.7603	2.25	1	2				
7	− 0.3754	2.77	3	2				
8	0.5317	0.88	2	1				
9	− 0.0354	2.71	2	2				
10	− 0.02 196	0.85	7	1				
11	− 0.0399	0.87	1		1.88	2.5	0.85	1
12	− 0.0112	1	2		25	900	1.08	0.93
13	− 0.4165	1.035	3		0.85	0.8	1.34	0.59
14	0.6293	0.95	2		0.81	0.79	1.33	0.73
15	− 0.03 702	2.26	1		0.86	1.3	1.38	0.56

The residual part of the Helmholtz energy considers intermolecular forces that lead to non-ideal behavior. Therefore, the residual part describes the deviation from ideal-gas behavior. While the ideal part is physically based, the residual part is an empirical formulation. For THF, the residual part consists of five monomial, five exponential, and five Gaussian bell-shaped terms:

9 $$\begin{aligned} \alpha ^{\text {r}}(\tau ,\delta )= & {} \sum _{i=1}^{5} n_i\delta ^{d_i}\tau ^{t_i}+\sum _{i=6}^{10} n_i\delta ^{d_i}\tau ^{t_i}\exp \left( -\delta ^{l_i}\right) \nonumber \\{} & {} +\sum _{i=11}^{15} n_i\delta ^{d_i}\tau ^{t_i}\exp \left( -\eta _i(\delta -\epsilon _i)^2 -\beta _i(\tau -\gamma _i)^2\right) \end{aligned}$$

The adjustable parameters $n_i$, $d_i$, $t_i$, $l_i$, $\eta _i$, $\epsilon _i$, $\beta _i$, and $\gamma _i$ are listed in Table 3. All thermodynamic properties can be calculated by combining derivatives of Eqs. 8 and 9 with respect to their independent variables. A detailed discussion about the calculation of thermodynamic properties with the EOS expressed in the Helmholtz energy is given by Span [11]. The new fundamental EOS is valid from the triple-point temperature of 164.67 K [22] to 550 K with pressures up to 600 MPa.

### Ancillary Equations

The determination of thermal saturation data requires the iterative calculation of saturation states by means of the Maxwell criterion [11]. The algorithm determines the vapor-liquid equilibrium by searching for liquid and vapor state points for a given temperature that has the same pressure and Gibbs energy in each phase. To aid in this iterative process, ancillary equations provide good estimations for the starting points.

The ancillary equations for vapor pressure $p_{\text{v}}$, saturated liquid density $\rho '$, and saturated vapor density $\rho ''$ use the following equations:

10 $$\begin{aligned} \ln \left( \frac{p_{\text {v}}}{p_{\text {c}}}\right)= & {} \frac{T_{\text {c}}}{T}\sum _{i=1}^{5}n_i\left( 1-\frac{T}{T_{\text {c}}}\right) ^{k_i} \end{aligned}$$

11 $$\begin{aligned} \frac{\rho '}{\rho _\text {c}}= & {} 1+\sum _{i=1}^{5}n_i\left( 1-\frac{T}{T_{\text {c}}}\right) ^{k_i} \end{aligned}$$

12 $$\begin{aligned} \ln \left( \frac{\rho ''}{\rho _{\text {c}}}\right)= & {} \sum _{i=1}^{6}n_i\left( 1-\frac{T}{T_{\text {c}}}\right) ^{k_i} \end{aligned}$$

Table 4 contains the values for the adjustable parameters $n_i$ and $k_i$. The critical parameters $T_\text {c}$, $p_\text {c}$, and $\rho _\text {c}$ are listed in Table 1.

**Table 4:** Parameters of the ancillary equations of the EOS for THF, cf. Equations 10 to 12

*i*	Vapor pressure $p_{\text{v}}$		Liquid density $\rho '$		Vapor density $\rho ''$	
	Eq. 10		Eq. 11		Eq. 12	
	$n_i$	$k_i$	$n_i$	$k_i$	$n_i$	$k_i$
1	− 7.82	1	6.9	0.5254	− 4.557	0.4897
2	4.1666	1.5	− 8.7784	0.782	− 8.9253	1.82
3	− 3.43	2	7.87	1.286	− 4.585	3.1
4	− 0.805	3.45	− 5.75	1.94	− 27.86	4.7
5	− 2.417	5	2.59	2.5	− 60.2	8.9
6	–	–	–	–	− 140	18

**Fig. 3:**
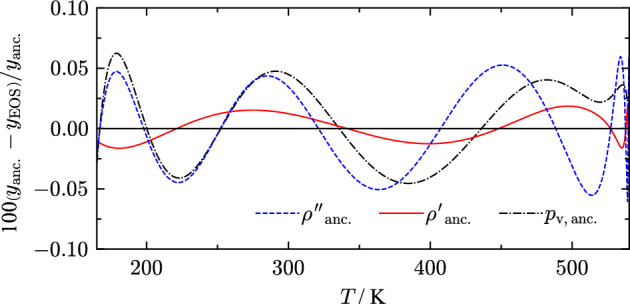
Percentage deviations of the ancillary equations for vapor pressure and saturation densities from values calculated with the present EOS

Deviations with respect to saturated liquid densities between the calculated values from Eq. 11 and values obtained by means of the Maxwell criterion [11] are less than 0.02 % over the entire temperature range, cf. Figure 3. Vapor pressures and saturated vapor densities (Eqs. 10 and 12) are represented within 0.06 %. However, these deviations are not uncertainties of saturation properties determined from the present EOS, which are discussed in Sect. 3.1.

## Validation of the EOS with Literature Data

The development of an equation of state is based on the underlying data base that includes measurements of various thermodynamic properties. The EOS is fitted to a carefully weighted selection of the most accurate data that will be discussed in this section. Derived from their agreement with the present EOS, uncertainties of the equation are estimated. For the statistical evaluation of all available data points, the relative deviation of each data point from the value determined with the EOS is calculated with

13 $$\begin{aligned} \frac{\Delta X}{X} = \frac{X_{\text {exp}}-X_{\text {calc}}}{X_{\text {exp}}}, \end{aligned}$$

where *X* is an arbitrary thermodynamic property. To assess the representation of each data set, the average absolute relative deviation (AARD) is used:

14 $$\begin{aligned} \text {AARD} = \frac{1}{n} \sum _{i=1}^{n}\left| \frac{\Delta X_i}{X_i}\right| , \end{aligned}$$

where *n* is the number of data points per publication. The AARD takes the deviations of each data point into account. Since deviations of a data set can vary depending on the represented fluid phase, deviations are separated into meaningful regions. The separation occurs differently for thermal saturation data compared to other types of data along the phase boundary. The measurements in each publication have been converted to molar-based SI units, with temperatures on the ITS-90 scale. [27]

### Vapor Pressure

**Fig. 4:**
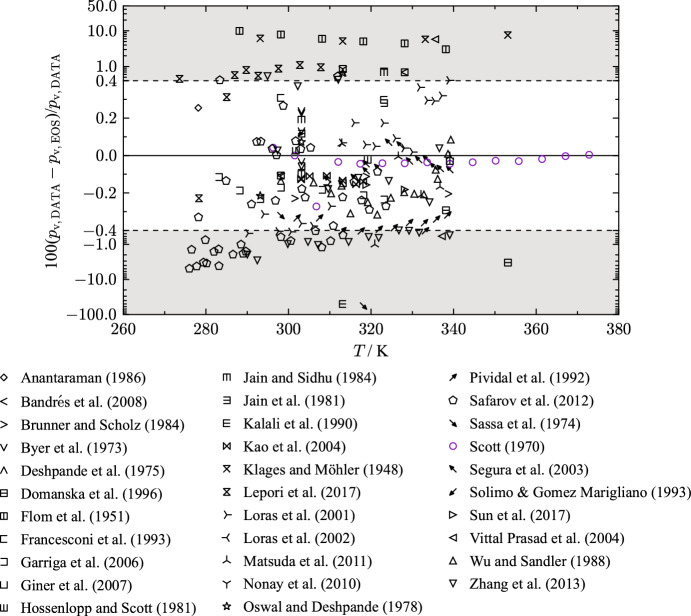
Percentage deviations of vapor-pressure data of selected authors from values calculated with the present EOS as a function of temperature. The ordinate is linearly scaled between the dashed lines and logarithmically scaled in the gray filled regions

The assessment of saturation state points is mostly restricted to vapor pressure measurements between 273 K and 340 K. Figure 4 provides an overview of all 34 available publications of vapor pressures for THF as percentage deviations as a function of temperature. A summary of the agreement of the present EOS with each publication is listed in Table 5. Due to the extent of publications, only the most accurate rated data sets are discussed in this section.

**Table 5:** Data summary and average absolute relative deviations (AARD) of experimental data for vapor pressure from the EOS

Reference	Year	No. of data	*T* (K)	AARD (%)			
				LT$^{\text{a}}$	MT$^{\text{a}}$	HT$^{\text{a}}$	Overall
Anantaraman [28]	1986	1	278.15	0.25	–	–	0.25
Bandrés et al. [29]	2008	2	298–329	0.11	0.69	–	0.40
Brunner and Scholz [30]	1984	3	301–339	0.17	0.20	–	0.18
Byer et al. [31]	1973	5	303.14	0.14	–	–	0.14
Deshpande et al. [32]	1975	2	298–314	0.40	–	–	0.40
Domanska et al. [33]	1996	2	338–354	–	1.8	–	1.8
Flom et al. [34]	1951	6	288–339	7.2	3.7	–	6.1
Francesconi et al. [35]	1993	1	298.15	0.31	–	–	0.31
Garriga et al. [36]	2006	9	283–324	0.17	–	–	0.17
Giner et al. [37]	2007	3	298–329	0.48	0.69	–	0.55
Giner et al. [38]	2007	3	298–329	0.48	0.69	–	0.55
Hossenlopp and Scott [24]	1981	3	301–340	0.025	0.029	–	0.026
Jain and Sidhu [39]	1984	4	303–324	0.42	–	–	0.42
Jain et al. [40]	1981	2	303–324	0.20	–	–	0.20
Kalali et al. [41]	1990	2	313–324	25	–	–	25
Kao et al. [42]	2004	7	303–319	0.13	–	–	0.13
Klages and Möhler [43]	1948	4	293–354	5.6	6.6	–	6.1
Kobe et al. [44]	1956	27	394–539	–	1.2	2.5	1.3
Lepori et al. [45]	2017	9	273–308	0.65	–	–	0.65
Loras et al. [46]	2001	20	290–339	0.27	0.23	–	0.25
Loras et al. [47]	2002	1	309.65	0.16	–	–	0.16
Matsuda et al. [48]	2011	6	312–340	0.54	0.098	–	0.25
Nonay et al. [49]	2010	2	298–314	0.48	–	–	0.48
Oswal and Deshpande [50]	1978	3	293–314	0.32	–	–	0.32
Pividal et al. [51]	1992	12	302–339	0.39	0.36	–	0.37
Safarov et al. [52]	2012	42	275–324	0.97	–	–	0.97
Sassa et al. [53]	1974	2	298–319	28	–	–	28
Scott [25]	1970	15	296–373	0.072	0.027	–	0.045
Segura et al. [54]	2003	13	318.82	0.10	0.05	–	0.073
Solimo and Gomez Marigliano [55]	1993	1	303.15	0.12	–	–	0.12
Sun et al. [56]	2017	2	318–329	0.16	0.18	–	0.17
Vittal Prasad et al. [57]	2004	2	335–338	–	3.2	–	3.2
Wu and Sandler [58]	1988	14	306–340	0.23	0.14	–	0.18
Zhang et al. [59]	2013	21	290–340	0.82	0.32	–	0.66

$^{\text{a}}$ LT: $T/T_{\text{c}} \le 0.6$, MT: $0.6 < T/T_{\text{c}} \le 0.98$, HT: $T/T_{\text{c}} \ge 0.98$

**Fig. 5:**
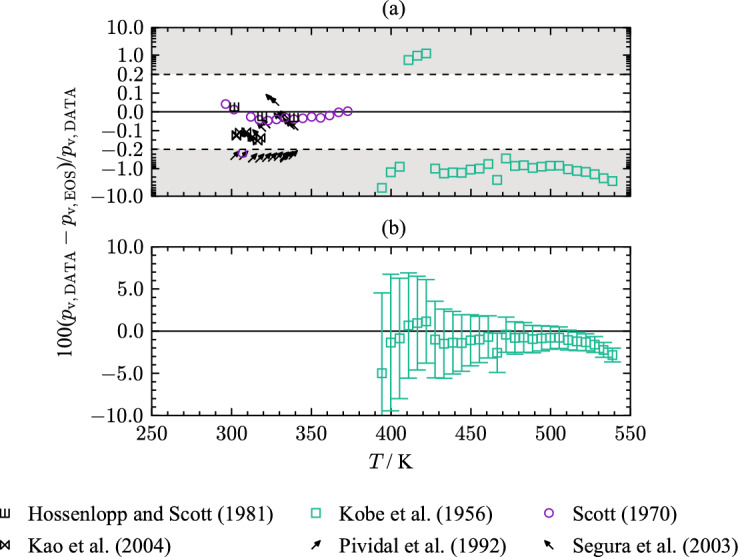
Top: Percentage deviations of vapor pressures of selected authors from values calculated with the present EOS as a function of temperature. The ordinate is linearly scaled between the dashed lines and logarithmically scaled in the gray filled regions. Bottom: Percentage deviations of vapor pressures of Kobe et al. [44] with error bars as a function of temperature calculated with the present EOS

In this regard, the top panel of Fig. 5 shows relative deviations of a data selection that will be presented in more detail.

Scott [25] measured 15 vapor pressure points in a temperature range from 296 K to 373 K. Measurements are obtained with a comparative ebulliometry, which is described in an earlier publication [60]. The authors state an uncertainty of 0.01 K in temperature and 0.0133 kPa in pressure [60]. Taking these uncertainties into account, an expanded combined uncertainty of 0.18 % (*k* = 2) is derived for the lowest temperature decreasing down to 0.053 % ($k = 2$) for the highest temperature. Figure 5 shows that the data set of Scott [25] includes one clear outlier at 306.75 K with a deviation of 0.27 %. Excluding this data point results in an AARD of 0.028 % (0.045 % with the outlier) with a maximum deviation 0.047 %. Therefore, all data points are represented within their uncertainty.

The vapor pressures measured by Hossenlopp and Scott [24] are well in line with the results of Scott [25]. Three data points in a temperature range from 301.8 K to 339.1 K have been determined multiple times in the process of measuring the heat of vaporization. The authors do not provide information regarding uncertainties of their measurements. With an AARD of 0.026 % and a maximum deviation of 0.029 %, the data align well with the present EOS.

Another data set was published by Kao et al. [42]. While investigating double azeotropy in binary mixtures, six vapor pressure points for THF in a temperature range of 303.05 K to 316.36 K were measured with an ebulliometer. The combined uncertainty of 0.1 % ($k = 2$) results from reported uncertainties of 0.01 K in temperature and 0.01 kPa in pressure. The top diagram of Fig. 5 shows an offset to the data of Scott [25]. The AARD of the data set of Kao et al. [42] is 0.13 % with a maximum deviation 0.15 %. The data are not represented within the assigned uncertainty of 0.1 %. However, the discrepancy to the present EOS is due to the offset from the data of Hossenlopp and Scott [24].

Segura et al. [54] published vapor pressures for THF measured with an ebulliometer. The measurements cover a temperature range from 319 K to 340 K. Experimental uncertainties of 0.02 K in temperature and 0.03 kPa in pressure are stated by the authors. Expanded uncertainties are estimated to 0.24 % ($k = 2$) at 319 K down to 0.14 % at 340 K. The top panel of Fig. 5 shows that the data are consistent with the results of Scott [25] while being represented within their experimental uncertainty.

Vapor-pressure data of Pividal et al. [51] have an uncertainty of 0.01 K in temperature and 0.01 kPa in pressure, which results in an expanded combined uncertainty of 0.1 % ($k= 2$) in pressure. As displayed in Fig. 5(a), the experimental data are represented with an offset between 0.3 % and 0.4 %. Considering the estimated uncertainties of the previous discussed publications, this data set does not align with vapor-pressure data of other authors, and, thus, cannot be described within the experimental uncertainty. The AARD of the measurements of Pividal et al. [51] is 0.37 %.

Vapor pressures for temperatures above 373 K up to 538.7 K have been measured by Kobe et al. [44]. In a study investigating critical properties and vapor pressures of ethers and heterocyclic compounds, 27 vapor pressures for THF were obtained. The authors assign a standard uncertainty of 2.07 kPa ($k = 1$) for the pressure measurement while stating that for some values a standard uncertainty of up to 20.7﻿ ﻿kPa ($k = 1$) is likely. In Fig. 5(b), the data of Kobe et al. [44] are displayed with error bars considering the expanded uncertainty of 41.4 kPa ($k = 2$). Up to 505 K, the present EOS agrees with the measurements within the combined uncertainty. In the vicinity of the critical temperature, the data deviate beyond the assigned uncertainties. Although the critical temperature of Kobe et al. [44] is consistent within 0.8 K, our derived critical pressure is about 2.2 % higher than the estimation of Kobe et al. [44]. The correct behavior of several thermodynamic properties in the critical region are considered in this work (see Sect. 4). In order to yield reasonable results in the critical region, the experimental data of Kobe et al. [44] could not be fitted within their stated uncertainty. Since the data of Kobe et al. [44] are the only available experimental data in the critical region, we could not validate their consistency with other experimental results and it is not clear if the error lies within the EOS or the data.

Considering the data comparison, the uncertainty of the EOS regarding vapor pressure is estimated to 0.05 % for temperatures between 296.3 K and 372 K. For temperatures above 372 K, the uncertainty is estimated to be 3 %.

### Heat of Vaporization

**Fig. 6:**
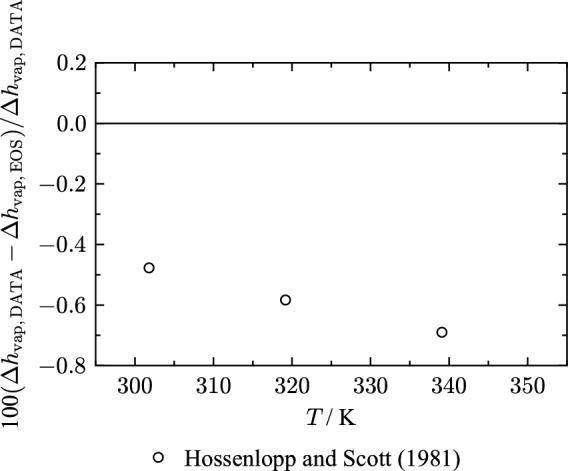
Percentage deviation of heat-of-vaporization data from values calculated with the present EOS as a function of temperature

A source for validating vapor pressure are heat-of-vaporization data $\Delta h_{\text{vap}}$ published by Hossenlopp and Scott [24]. To determine the heat of vaporization, the amount of electrical energy is measured that is required to vaporize a given amount of fluid [61]. The authors assign an expanded uncertainty of 0.2 % ($k = 2$). The data is represented with an AARD of 0.58 % and are, therefore, not described within their estimated uncertainty. A detailed overview of the AARD is given in Table 6. Figure 6 shows that the data exhibit an offset to the EOS. The data set of Hossenlopp and Scott [24] was taken into consideration during the regression process.

**Table 6:** Data summary and average absolute relative deviations (AARD) of experimental data for heat of vaporization from the EOS

Reference	Year	No. of data	*T* (K)	AARD (%)			
				LT$^{\text{a}}$	MT$^{\text{a}}$	HT$^{\text{a}}$	Overall
Hossenlopp and Scott [24]	1981	3	301.8-$-$339.1	0.53	0.69	–	0.58

$^{\text{a}}$ LT: $T/T_{\text{c}} \le 0.6$, MT: $0.6 < T/T_{\text{c}} \le 0.98$, HT: $T/T_{\text{c}} \ge 0.98$

The uncertainty of the present EOS regarding heat of vaporization in the temperature range of 300 K to 340 K is estimated to be 0.8 %.

### Homogeneous Density

All density data were measured in the liquid phase with most experimental studies being carried out at atmospheric pressures. Due to the extent of available density measurements at atmospheric pressure, only the studies with a significant amount of data points or measurements at elevated pressures are discussed in this section. Table 7 lists available publications, including the AARD to the present EOS in the liquid phase, that comprise more than two data points. An overview of all collected $p\rho T$ publications ($n_{\text{Data}} \le 2$: [28, 29, 35, 37, 41, 47, 48, 54, 55, 57, 62–133]) for THF is provided in the supplementary material. Deviation diagrams including all collected density measurements are shown as well.

**Table 7:** Data summary and average absolute relative deviations (AARD) of experimental data for homogeneous densities in the liquid phase from the EOS

Reference	Year	No. of data	*T* (K)	*p* (MPa)	AARD (%)
Afanasyev and Zyatkova [134]	1996	3	283–314	0.101 325	0.19
Almasi [135]	2018	4	298–314	0.101 325	0.014
Aminabhavi et al. [136]	1989	3	298–319	0.101 325	0.77
Aminabhavi and Patil [137]	1998	3	298–309	0.101 325	2.3
Aralaguppi et al. [138]	1996	3	298–309	0.101 325	0.090
Back and Woolf [139]	1998	45	278–324	0.1–300	0.056
Belandria et al. [140]	2009	9	293–334	0.101 325	0.87
Brocos et al. [141]	1996	3	288–309	0.101 325	0.009
Carvajal et al. [142]	1965	10	203–299	0.101 325	0.61
Chen et al. [143]	2015	7	293–324	0.101 325	0.041
Choudhury et al. [144]	2003	5	303–323	0.101 325	0.22
Comelli and Francesconi [145]	1991	10	290–304	0.101 325	0.19
Das et al. [146]	1994	3	298–318	0.101 325	0.14
Das and Roy [147]	2006	3	298–319	0.101 325	0.11
Dhaduk et al. [148]	2015	4	298–313	0.101 325	0.030
Fattahi and Iloukhani [149]	2010	3	288–309	0.101 325	0.005
Gadžurić et al. [150]	2012	5	293–314	0.101 325	0.13
Giner et al. [38]	2007	3	298–329	0.101 325	0.015
Govender et al. [151]	1996	50	288–329	0.1–8.0	0.56
Holland and Smyth [152]	1955	3	274–314	0.101 325	0.14
Ijardar and Malek [153]	2014	7	293–324	0.101 325	0.090
Inglese et al. [154]	1983	3	298–319	0.101 325	0.11
Ivanov [155]	2011	5	278–319	0.101 325	0.008
Ivanov [156]	2014	9	278–319	0.101 325	0.006
Jatkar and Deshpande [157]	1960	7	298–329	0.101 325	0.14
Jha et al. [158]	2003	6	298–323	0.101 325	0.21
Kinart et al. [159]	2002	5	291–309	0.101 325	0.004
Klages and Möhler [43]	1948	4	293–354	0.101 325	0.19
Kneževic-Stevanovic et al. [160]	2013	8	288–324	0.101 325	0.74
Ku et al. [161]	2008	3	288–309	0.101 325	0.031
Kumar [162]	2000	6	288–339	0.101 325	1.8
Marczak et al. [163]	2008	5	297–314	0.101 325	0.023
Mariano et al. [164]	2000	3	283–314	0.101 325	0.050
Muhuri et al. [165]	1996	3	298–319	0.101 325	0.14
Nain [166]	2006	9	278–319	0.101 325	0.14
Nain and Droliya [167]	2017	6	293–319	0.101 325	0.13
Nayak et al. [168]	2003	3	298–309	0.101 325	0.082
Nayak et al. [169]	2004	3	303–324	0.101 325	0.025
Nicolas et al. [170]	1980	7	223–293	0.101 325	0.32
Nikolic et al. [171]	2005	4	303–319	0.101 325	0.051
Nikolic et al. [172]	2006	4	303–319	0.101 325	0.051
Nonay et al. [49]	2010	3	283–314	0.101 325	0.017
Oswal et al. [173]	2010	3	303–324	0.101 325	0.20
Ottani et al. [174]	2002	6	297–308	0.101 325	0.015
Ottani et al. [175]	2003	3	288–314	0.101 325	0.018
Pérez et al. [176]	2003	3	283–314	0.101 325	0.038
Palani and Geetha [177]	2009	3	303–313	0.101 325	0.19
Pandiyan et al. [178]	2011	3	303–324	0.101 325	0.058
Piñeiro et al. [179]	2002	11	293–304	0.101 325	0.006
Postigo et al. [180]	2003	3	283–314	0.101 325	0.050
Ramkumar and Kudchadker [181]	1989	5	278–299	0.101 325	0.024
Rathnam et al. [182]	2013	4	298–314	0.101 325	0.18
Rathnam et al. [183]	2013	3	303–314	0.101 325	0.22
Rodnikova et al. [184]	2011	5	293–334	0.101 325	0.008
Roy et al. [185]	2001	5	298–318	0.101 325	0.10
Saleh et al. [186]	2002	5	303–324	0.101 325	0.53
Schedemann [187]	2009	459	283–443	0.3–130	0.12
Schornack and Eckert [188]	1970	14	303–324	0.1–517	1.5
Shelar et al. [189]	2016	4	298–313	0.101 325	0.26
Sinha et al. [190]	2013	3	298–319	0.101 325	0.12
Sinha and Roy [191]	2006	3	298–319	0.101 325	0.12
Sinha and Roy [192]	2006	3	303–324	0.101 325	0.23
Torres et al. [193]	2008	4	288–304	0.101 325	0.006
Vaid et al. [194]	2015	7	293–324	0.101 325	0.15
Valén et al. [195]	2002	3	283–314	0.101 325	0.004
Valén et al. [196]	2002	3	283–314	0.101 325	0.004
Valen et al. [197]	2003	3	283–314	0.101 325	0.004
Vercher et al. [198]	2011	5	278–319	0.1	0.007
Wankhede et al. [199]	2008	3	288–309	0.101 325	0.006
Wankhede et al. [200]	2010	3	288–304	0.101 325	0.22
Živkovic et al. [201]	2014	8	288–324	0.101 325	0.049

Clear outliers were not considered in the calculation of the AARD

**Fig. 7:**
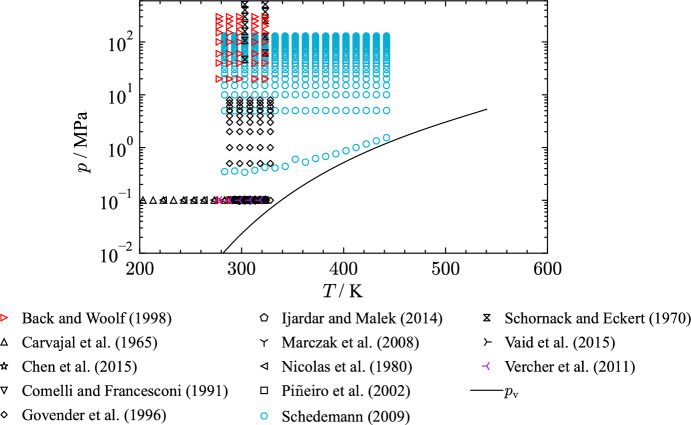
*p*,*T* diagram of THF showing the distribution of density measurements from selected authors

**Fig. 8:**
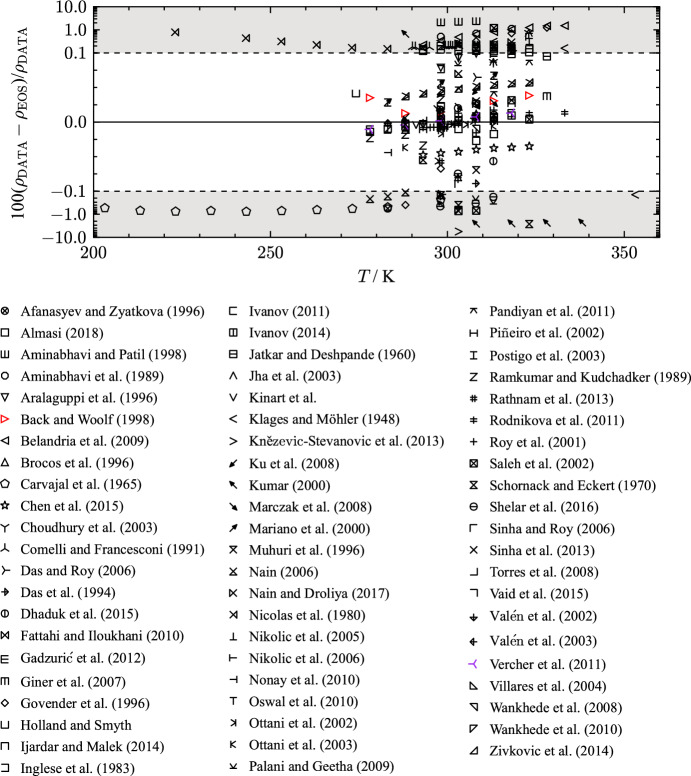
Percentage deviations of density data of selected authors as a function of temperature calculated with the present EOS at atmospheric pressure. The ordinate is linearly scaled between the dashed lines and logarithmically scaled in the gray filled regions

Figure 7 shows the location of selected density measurements in a *p*,*T*-diagram. The data cover a temperature range from 200 K up to 440 K at pressures between 0.1 MPa to 600 MPa. The majority of density measurements were performed at atmospheric pressure. Figure 8 shows deviations in density over temperature at atmospheric pressure of selected publications.

The most accurate source for density measurements at atmospheric pressure at ambient temperatures is the experimental study of Vercher et al. [198]. The authors assign uncertainties of 0.001 K for temperature and 0.007 ${{\text{kg}}\cdot{\text{m}}^{-3}}$ for density measurements. However, the uncertainty estimation of the density measurements seems optimistic. For Anton Paar DSA apparatuses, Prokopová et al. [202] recommend uncertainties of at least 0.03 ${\text{kg}}{\cdot}{\text{m}}^{-3}$ for low-viscosity fluids at atmospheric pressure. Without considering impurities, an expanded combined uncertainty of 0.007 % ($k = 2$) is assigned. Due to the low uncertainty estimated by the authors and the alignment with the data of Back and Woolf [139], Piñeiro et al. [179], and the density measurements along the lowest isotherm of Schedemann [187], the data of Vercher et al. [198] were chosen for the regression process. The AARD of this data set is 0.007 % with a maximum deviation of 0.01 %. The data are not described by the new EOS within the assigned uncertainty and, yet, the EOS is in very good agreement with this data set.

Several other authors [148, 156, 179] used the same vibrating-tube apparatus and obtained consistent results with the measurements of Vercher et al. [198]. The main contributor to the uncertainty estimate of these apparatuses is the recommended value of at least 0.03 ${\text{kg}}{\cdot}{\text{m}}^{-3}$ from Prokopová et al. [202] that was applied to the data of Dhaduk et al. [148], Ivanov [156], and Piñeiro et al. [179]. Taking the uncertainty in temperature into account, the combined uncertainty is estimated to be 0.007 % ($k = 2$) for these three publications, which is the same valuation as for Vercher et al. [198].

**Fig. 9:**
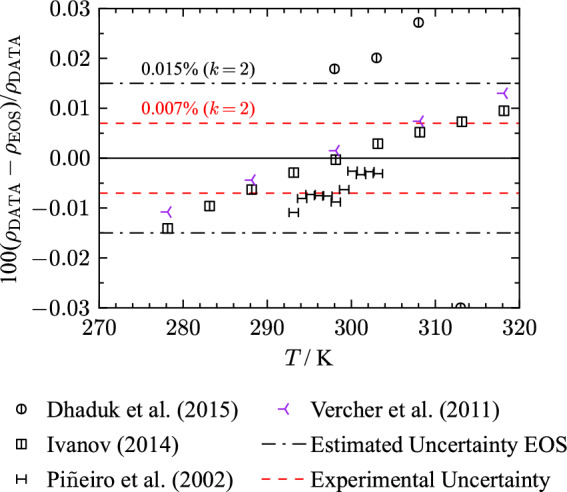
Percentage deviations of density data of selected authors as a function of temperature calculated with the present EOS at atmospheric pressure. The displayed density measurements have an expanded combined uncertainty of 0.007 % ($k = 2$)

Figure 9 displays percentage deviations of the introduced publications that measured with a vibrating-tube densimeter. The dashed line corresponds to the estimated experimental uncertainty of 0.007 % ($k = 2$). The experimental data of Ivanov [156] are in very good agreement with measurements of Vercher et al. [198]. Piñeiro et al. [179] measured slightly lower densities at corresponding temperatures but the data still align with Ivanov [156] and Vercher et al. [198] within the experimental uncertainty. The results obtained by Dhaduk et al. [148] have an offset of 0.02 % to the other publications including one clear outlier at 313 K.

The present EOS cannot reproduce the discussed data within their experimental uncertainty. Figure 9 indicates a temperature dependent deviation of the EOS to the experimental data that causes the maximum deviations of all data sets to be outside their experimental uncertainty. Nonetheless, the alignment with the discussed density data allows for an uncertainty estimation for homogeneous liquid densities of 0.015 % ($k= 2$) for the present EOS, see Fig. 9.

Comelli and Francesconi [145] investigated the density of THF with a two-capillary glass pycnometer in a temperature range from 290 K to 304 K. They estimated the uncertainty in density to be 0.2 ${\text{mg}}{\cdot}{\text{cm}}^{-3}$. With the given standard uncertainty by the authors, an expanded uncertainty of 0.05 % ($k = 2$) is calculated; although, the experimental data have a systematic offset of 0.2 % compared to experimental results of Vercher et al. [198]. The calculated AARD is 0.19 % and the maximum deviation is 0.2 %.

Below 273.15 K, two experimental studies of Carvajal et al. [142] and Nicolas et al. [170] provide density measurements at atmospheric pressure. Carvajal et al. [142] measured density, viscosity, and dielectric constants of dimethoxyethane and THF for temperatures between 203 K and 299 K at ambient pressure. The authors assign an expanded uncertainty of 1 % ($k = 2$). With an AARD of 0.61 % and a maximum deviation of 0.76 %, the data are represented within the experimental uncertainty by the present EOS.

In the course of a study focusing on dielectric constants of methanol, THF, and their mixtures, Nicolas et al. [170] measured densities of pure THF. Molar volumes of a THF sample with a purity of 99.9 mass % were measured in a temperature range of 223 K to 293 K at ambient pressure. The authors do not state information about the uncertainty of their measurements. The AARD of the data set is 0.32 % with a maximum deviation of 0.79 %.

**Fig. 10:**
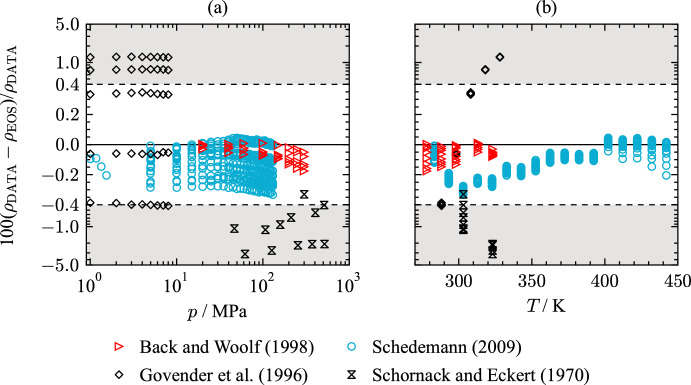
Percentage deviations of density data of selected authors from values calculated with the present EOS as a function of pressure (a) and as a function of temperature (b). The ordinate is linearly scaled between the dashed lines and logarithmically scaled in the gray filled regions

Available literature data at pressures above atmospheric conditions are sparse. Figure 10 displays percentage deviations of all available density data above atmospheric pressure. The most comprehensive study was published by Schedemann [187]. Measurements were carried out with a vibrating-tube densimeter in a pressure range of 0.34 MPa to 140 MPa and a temperature range from 263 K to 473 K. Considering the uncertainties in pressure, density, and temperature, a combined uncertainty of 0.2 % ($k = 2$) is assigned to this data set. As shown in Fig. 7, the publication of Schedemann [203] is the only source providing density data at temperatures above 328.15 K. At the lowest isotherm of 283 K, the data are well in line with other data sets. With increasing temperature, deviations from the present EOS increase. Due to the lack of other available $p\rho T$ measurements in this temperature range, the quality of the data is not assessable. The AARD of the data set is 0.12 % with a maximum deviation of 0.33 %. Figure 10(b) displays temperature dependent deviations of the experimental results. In the temperature range between 290 K and 340 K, the data are not represented within their experimental uncertainty. At these conditions, however, the data of Schedemann [203] are contrary to the density measurements of Back and Woolf [139], which were considered more accurate in this temperature region. This results in an offset of Schedemann’s [203] data to the present EOS.

Govender et al. [151] provide the second largest set of $p\rho T$ measurements. In a pressure range from 0.1 MPa to 8 MPa, the authors performed measurements along 5 isotherms between 288.15 K and 328.15 K. Taking the stated uncertainties of 0.03 K in temperature, 0.01 MPa in pressure, and 0.03 ${\text{kg}}{\cdot}{\text{m}}^{-3}$ in density into account, an expanded combined uncertainty of 0.01 % ($k = 2$) was assigned to this data set. With an AARD of 0.56 % and a maximum deviation of 1.175 %, the data are not described within the stated uncertainties of the authors. Figure 10(b) shows temperature dependent deviations in the data of Govender et al. [151]. Only at the 298.15 K isotherm, the density measurements are in line with the other reported density data.

Another important set of density data was measured by Back and Woolf [139] during an experimental study focusing on mixtures of water and THF. Density measurements of pure THF were carried out at 45 state points in the temperature range from 278.15 K to 323.15 K and pressures between 0.1 MPa and 300 MPa. The authors provide a temperature uncertainty of 0.01 K and 0.05 % with respect to pressure. The expected uncertainties in density measurements were given in an earlier work [204]. Depending on the state region, the expanded uncertainty of the $p\rho T$ data was calculated to be between 0.06 % and 0.4 % ($k = 2$). At atmospheric pressure (see Fig. 8), experimental data of Back and Woolf [139] are represented within 0.04 % with the present EOS, and therewith, show good agreement with other publications at atmospheric pressure. The AARD of the data is 0.056 % with a maximum deviation of 0.18 %. At pressures above 150 MPa and temperatures below 300 K, the presented EOS does not match the stated uncertainties of the authors. Yet, the majority of the data is represented within their uncertainties, and all data are well in line with the present EOS.

Density measurements of Schornack and Eckert [188] also cover the high pressure region along two isotherms of 303.15 K and 323.15 K. The authors state an expanded combined uncertainty of 0.15 % ($k = 2$) in density. However, Fig. 10 shows that the data are not in line with each other, neither at atmospheric pressure, nor at high pressures. The density measurements deviate between 0.3 % up to 3.1 % from the present EOS.

Based on the comparison with the available literature data, the uncertainty in density of the present EOS can be estimated. At atmospheric pressure and temperatures between 278 K and 320 K, the uncertainty of the EOS is 0.015 %. The lower temperature range at ambient pressure is represented with an uncertainty of 0.7 %. Calculations of densities at pressures above 0.1 MPa have an uncertainty of 0.2 % between 278 K and 443 K. At temperatures and pressures outside the available literature data, an uncertainty estimation is not possible.

### Second Virial Coefficient

Thermal properties of the gaseous phase can also be assessed with the help of virial coefficients. These can be determined from statistical mechanics, by direct measurements, or by extrapolation of gaseous densities or gaseous speed of sound data, where the virial expansion is mostly truncated after the third (*C*) or even the second (*B*) virial coefficient. For THF, available data for virial coefficients are limited to the second virial coefficient *B*. Because of the lack of accurate data derived from measurements, some simple correlations for *B* were taken into consideration. The available literature data are listed in Table 8 including the average absolute relative deviation calculated with the present EOS. Relative deviations are considered since the with data for *B* covered temperature range is much lower than the Boyle temperature of THF ($T_{\text{BL}} = 1228$ K).

**Table 8:** Data summary and average absolute relative deviations (AARD) of second virial coefficient data from the EOS

Reference	Year	No. of data	*T* (K)	AARD (%)
Hossenlopp and Scott [24]	1981	3	301–340	22.8
Jain and Sidhu [39]	1984	2	303–324	13.1
Jain et al. [40]	1981	1	323.14	12.6
Nonay et al. [49]	2010	1	298.15	12.1
Oswal and Deshpande [50]	1978	3	293–314	13.5

**Fig. 11:**
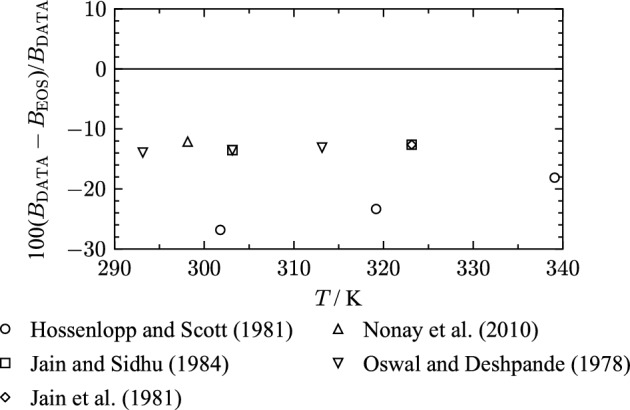
Percentage deviations of second virial-coefficient data as a function of temperature from the present EOS

The only publication that correlates values for *B* from experimental data is the study of Hossenlopp and Scott [24] that has already been described in Sect. 3.1. *B* is determined by combining the virial equation with the following Clapeyron equation:

15 $$\begin{aligned} B = \left( \frac{\Delta h_{\text{vap}}}{T\frac{\partial p_{\text{v}}}{\partial T}} + v_{\text{L}}\right) \cdot \left( \frac{p \left( \frac{\Delta h_{\text{vap}}}{T\frac{\partial p_{\text{v}}}{\partial T}} + v_{\text{L}}\right) }{RT} -1 \right) , \end{aligned}$$

with the temperature derivative of the vapor pressure $\frac{\partial p_{\text{v}}}{\partial T}$, and the molar volume of the liquid $v_{\text{L}}$. The experimental values for $\Delta h_{\text{vap}}$ from the same publication presented in Sect. 3.2 were used. $v_{\text{L}}$ was calculated from density data, which were not specified by the authors. For $\frac{\partial p_{\text{v}}}{\partial T}$, literature data were used. As shown in Fig. 11, the data are represented within 30 % by the present EOS. In order to quantify the capability of the correlation for *B*, Eq. 15 was used with values for $\frac{\partial p_{\text{v}}}{\partial T}$, $\Delta h_{\text{vap}}$, and $v_{\text{L}}$ calculated with the present EOS. Resulting values for the second virial coefficient *B* from the correlation in Eq. 15 agree with calculated values for *B* with the EOS within 2 %. Thus, we conclude the uncertainty of this methodology to be 2 %. During the regression process, we found that the calculated second virial coefficients of Hossenlopp and Scott [24] are not consistent with vapor-pressure data. The inconsistency might result from inappropriate values for the temperature derivative of the vapor pressure $\frac{\partial p_{\text{v}}}{\partial T}$, which Hossenlopp and Scott [24] obtained from the literature (see Sect. 3.1). Hence, the focus was set on good representation of available vapor-pressure data. Further, the significant deviations might result from unknown used values of $v_{\text{L}}$ and deviations in $\Delta h_{\text{vap}}$ from the present EOS (see Sect. 3.2).

In three publications [39, 40, 50], second virial coefficients were calculated with Berthelot’s equation [205]

16 $$\begin{aligned} B=\frac{9}{128} \frac{RT_{\text{c}}}{p_{\text{c}}} \left( 1 - \frac{T_{\text{c}}^2}{T^2} \right) , \end{aligned}$$

with the critical parameters $T_{\text{c}}$ and $p_{\text{c}}$. Oswal and Deshpande [50] used $T_{\text{c}} = 540.2$ K and $p_{\text{c}} = 5.32$ MPa [206] in Eq. 16. Jain et al. [40] and Jain and Sidhu [39] do not provide information regarding the applied critical parameters. Due to the same estimation method for values of *B*, the three data sets [39, 40, 50] are very consistent. Since all these data sets use a generalized expression for *B*, they were not taken into consideration. In the publication of Nonay et al. [49], the provided value for *B* cannot be reproduced from the given reference, and was therefore also not taken into account.

The available literature data for the second virial coefficient does not allow for a resilient estimation of the uncertainty of the present EOS regarding calculated values for the second virial coefficient *B*. However, the qualitative course of virial coefficients is evaluated in Sect. 4.

### Speed of Sound

For developing an EOS, caloric data, e.g., speed of sound (*w*), are fundamental for assessing the overall functional form of the equation. Other than $p\rho T$ correlations, which only depend on the first derivative of the residual Helmholtz energy with respect to density, caloric properties, such as speed of sound and heat capacities, are calculated with higher-order derivatives including the ideal and residual parts of the equation as well as temperature derivatives. Speed of sound measurements can be determined with high accuracy with modern measurement techniques. Thus, their precise representation is crucial for the performance of the EOS.

**Fig. 12:**
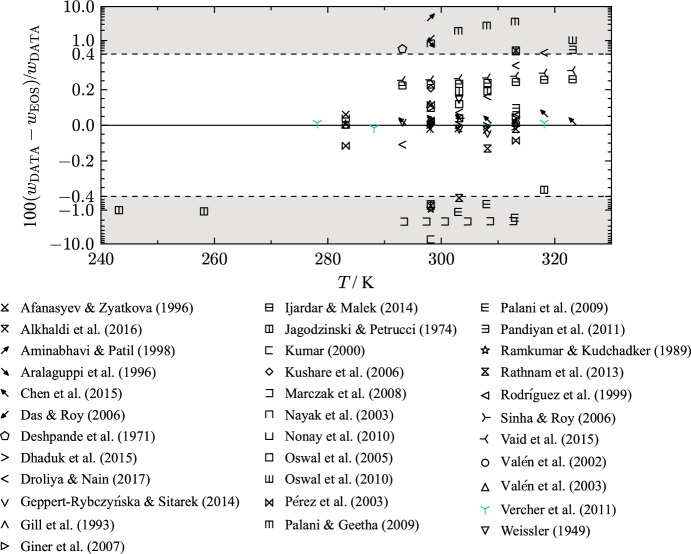
Percentage relative deviation of speed-of-sound data at atmospheric pressure as a function of temperature calculated with the present EOS. The ordinate is linearly scaled between the dashed lines and logarithmically scaled in the gray filled regions

For THF, the available experimental data for speed of sound is limited to atmospheric pressure where the data cover a temperature range from 243 K to 324 K. Figure 12 displays percentage deviations in speed of sound of all available literature data. An overview of the experimental data including the AARD is given in Table 9. Vaid et al. [194], Ijardar and Malek [153], Chen et al. [143], Dhaduk et al. [148], and Vercher et al. [198] performed speed of sound measurements with the same measurement apparatus (tube densimeter Anton Paar DSA 5000). It has a build in solid-state thermostat and is capable of simultaneous measurement of density and speed of sound.

**Table 9:** Data summary and average absolute relative deviations (AARD) of speed of sound data in the liquid phase from the EOS

Reference	Year	No. of data	*T* (K)	*p* (MPa)	AARD (%)
Afanasyev and Zyatkova [134]	1996	3	283–314	0.101 325	0.036
Alkhaldi et al. [207]	2016	3	298–308	0.101 325	0.022
Aminabhavi and Patil [137]	1998	1	298.15	0.101 325	4.8
Aralaguppi et al. [138]	1996	1	298.15	0.101 325	0.81
Chen et al. [143]	2015	7	293–324	0.101 325	0.033
Das and Roy [147]	2006	1	298.15	0.101 325	1.1
Deshpande et al. [73]	1971	1	293.14	0.101 325	0.56
Dhaduk et al. [148]	2015	4	298–313	0.101 325	0.004
Droliya and Nain [208]	2017	6	293–319	0.101 325	0.19
Geppert-Rybczyńska and Sitarek [64]	2014	4	293–309	0.1	0.023
Gill et al. [81]	1993	1	298.00	0.101 325	0.12
Giner et al. [209]	2007	2	298–314	0.101 325	0.072
Ijardar and Malek [153]	2014	7	293–324	0.101 325	0.24
Jagodzinski and Petrucci [86]	1974	5	243–319	0.101 325	0.67
Kumar [162]	2000	1	298.15	0.101 325	7.4
Kushare et al. [92]	2006	2	298.15	0.101 325	0.21
Marczak et al. [163]	2008	6	293–313	0.101 325	2.2
Nayak et al. [168]	2003	1	298.15	0.101 325	0.81
Nonay et al. [49]	2010	2	283–299	0.101 325	0.069
Oswal et al. [102]	2005	2	303.15	0.101 325	0.12
Oswal et al. [173]	2010	3	303–324	0.101 325	0.57
Palani and Geetha [177]	2009	3	303–313	0.101 325	2.7
Palani et al. [131]	2009	3	303–313	0.101 325	1.2
Pandiyan et al. [178]	2011	3	303–324	0.101 325	0.22
Pérez et al. [176]	2003	3	283–314	0.101 325	0.10
Ramkumar and Kudchadker [181]	1989	1	298.14	0.101 325	0.91
Rathnam et al. [183]	2013	4	298–314	0.101 325	0.46
Rodríguez et al. [132]	1999	2	298–314	0.101 325	0.037
Sinha and Roy [192]	2006	1	303.15	0.101 325	0.039
Vaid et al. [194]	2015	7	293–324	0.1	0.27
Valén et al. [196]	2002	6	283–314	0.101 325	0.012
Valén et al. [195]	2002	3	283–314	0.101 325	0.012
Valén et al. [197]	2003	3	283–314	0.101 325	0.012
Vercher et al. [198]	2011	5	278–319	0.1	0.011
Weissler [120]	1949	1	303.13	0.101 325	0.14

Clear outliers were not considered in the calculation of the AARD

**Fig. 13:**
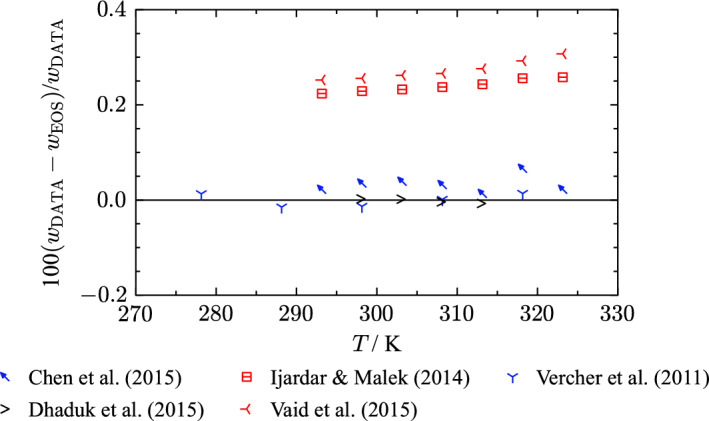
Relative deviations in speed-of-sound data as a function of temperature calculated with the present EOS. Studies with mass fraction purities $< 99.5$ % are marked red and studies with mass fraction purities $\ge 99.5$ % are marked blue. Black corresponds to studies with unknown sample purity

Vaid et al. [194] carried out measurements at 0.1007 MPa with a standard uncertainty of 0.5 kPa ($k = 1$). The standard uncertainties of the temperature and the speed of sound measurement are stated to be 0.05 K ($k = 1$) and 0.5 ${\text{m}}{\cdot}{\text{s}}^{-1}$ ($k = 1$). Overall, an expanded combined uncertainty of 0.1 % ($k = 2$) is assigned. In a previous study of Ijardar and Malek [153], the same working group as Vaid et al. [194] investigated speed of sound of THF. Here, the authors state lower standard uncertainties in temperature and speed of sound than in the later publication, 0.01 K ($k = 1$) and 0.01 ${\text{m}}{\cdot}{\text{s}}^{-1}$ ($k = 1$). We found no differences in the measurement method, sample preparation, and calibration between the two publications. Therefore, the expanded uncertainty of Vaid et al. [194] is also applied to the results of Ijardar and Malek [153], which is 0.1 % ($k = 2$). Chen et al. [143] measured speed of sound and density of pure THF at atmospheric pressure in a temperature range from 293 K to 324 K. The authors assign a standard uncertainty of 2 ${\text{m}}{\cdot}{\text{s}}^{-1}$ ($k = 1$) in speed of sound, and 0.01 K ($k = 1$) in temperature. An expanded combined uncertainty of 0.35 % ($k = 2$) is assigned to the data. The fourth study with this apparatus was published by Vercher et al. [198]. Standard uncertainties of 0.001 MPa ($k = 1$) in pressure, 0.001 K ($k = 1$) in temperature and 0.05 ${\text{m}}{\cdot}{\text{s}}^{-1}$ ($k = 1$) in speed of sound are stated by the authors, which is a significant lower uncertainty estimation than in previously described studies [143, 153, 194]. Similar to the measured densities of Vercher et al. [198] (see Sect. 3.3), the estimated uncertainty in speed of sound cannot be verified and seems optimistic. It is likely that Vercher et al. [198] do not refer to the uncertainty of their results but to the repeatability. Fortin et al. [210] provide a very detailed uncertainty estimation for speed of sound measurement carried out on the same apparatus. The manufacturers uncertainty specification for the vibrating-tube densimeter is 0.5 ${\text{m}}{\cdot}{\text{s}}^{-1}$, which is one order of magnitude higher than the value of Vercher et al. [198]. After calibration and validation, Fortin et al. [210] state an expanded uncertainty between 0.4 ${\text{m}}{\cdot}{\text{s}}^{-1}$ ($k = 2$) and 0.6 ${\text{m}}{\cdot}{\text{s}}^{-1}$ ($k = 2$), which translates to an expanded combined uncertainty of at least 0.03 % ($k = 2$). Therewith, we conclude that an expanded combined uncertainty of 0.03 % ($k = 2$) is a more reasonable estimation for the measurements of Vercher et al. [198]. Further measurements of speed of sound performed on the apparatus of the same manufacturer were carried out by Dhaduk et al. [148]. With the provided standard uncertainties of 0.002 K ($k = 1$) in temperature and 0.13 ${\text{m}}{\cdot}{\text{s}}^{-1}$ ($k = 1$) in speed of sound, an overall expanded uncertainty of 0.011 % ($k = 2$) was predicted. Although the present EOS represents all data points within 0.007 %, a combined expanded uncertainty of 0.03 % ($k = 2$) is a more realistic estimation based on the discussion of Fortin et al. [210].

Figure 12 shows that experimental results reported by the working group of Vaid et al. [153, 194] are consistent with data from their laboratory but show an offset of at least 0.2 % to other publications that used the same measurement apparatus [143, 148, 198]. It is likely that the offset is a result of sample impurities or due to calibration. Figure 13 illustrates these publications colored according to the purity of the THF sample. Out of the described publications, Vaid et al. [194] and Ijardar and Malek [153] investigated THF samples with the highest amount of impurity, 0.7 mass %. The density data with lower impurity content exhibit good agreement within their classification of a mass impurity fraction $\le 0.5$ %. Thus, we found the experimental results of Chen et al. [143], Vercher et al. [198], and Dhaduk et al. [148] to be more trustworthy. It is also possible that an inappropriate calibration induced an offset of such magnitude. Whether the offset of Vaid et al. [194] and Ijardar and Malek [153] is due to impurities or an incorrect calibration cannot be definitely identified.

Further comparison to speed of sound measurements carried out with different measurement techniques support these assumptions.

**Fig. 14:**
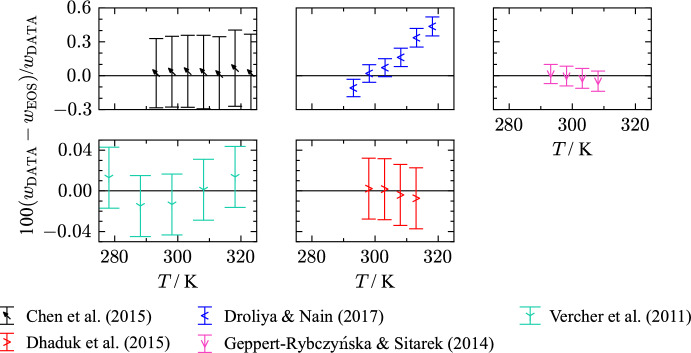
Percentage relative deviations of speed-of-sound data of selected authors at atmospheric pressure with experimental uncertainties as a function of temperature

Geppert-Rybczyńska and Sitarek [64] performed speed of sound measurements of THF on a pulse-echo overlap meter. The standard uncertainties are stated to be 0.01 K ($k = 1$) in temperature and 0.5 ${\text{m}}{\cdot}{\text{s}}^{-1}$ ($k = 1$) in speed of sound. The fluid sample had a mass fraction purity of 99.9 mass %. Overall, an expanded combined uncertainty of 0.09 % ($k = 2$) was determined, see Fig. 14. The experimental results of Geppert-Rybczyńska and Sitarek [64] are in good agreement with previous introduced investigations. The speed of sound data align with the measurements of Chen et al. [143], Vercher et al. [198], and Dhaduk et al. [148], and are represented with their experimental uncertainty by the present EOS.

Droliya and Nain [208] used a single-crystal variable-path multifrequency ultrasonic interferometer to measure speed of sound of a THF sample with a mass fraction purity of 99.7 mass %. The authors assign a standard uncertainty of 0.5 ${\text{m}}{\cdot}{\text{s}}^{-1}$ ($k = 1$) to the speed of sound measurement and 0.01 K ($k = 1$) in temperature. A combined uncertainty of 0.09 % ($k = 2$) is assigned. With a maximum deviation of 0.44 %, the present EOS does not describe all data points within their stated uncertainties. Figure 14 shows a temperature dependent shift of the data compared to other accurate *w* measurements. A possible explanation is an incorrect calibration. We found that temperature dependent deviations are also present for other investigated fluids. e.g., in the study of Droliya and Nain [208], which is an indication of a systematic error.

The only publication that provides *w* data for temperatures below 278.15 K was published by Jagodzinski and Petrucci [86]. The study covers a temperature range between 243 K and 318 K with a given uncertainty in temperature of 0.05 K. For the speed of sound measurement, Jagodzinski and Petrucci [211] estimate a maximum standard uncertainty of 19 ${\text{m}}{\cdot}{\text{s}}^{-1}$ ($k = 1$). Thus, an expanded combined uncertainty of 2.5 % ($k = 2$) is assigned. The AARD of this data set is 0.67 % with a maximum deviation of 1.15 %. Therefore, the present EOS describes the data within the experimental uncertainty.

Taking the available literature data into account, the uncertainty of the present EOS regarding speed of sound at ambient pressure for temperatures between 243 K and 275 K is estimated to 1.5 %. In the temperature range from 275 K to 320 K, an uncertainty of 0.03 % is assigned. Outside these temperature and pressure ranges, no definite estimation of the uncertainty of the present EOS is possible.

### Isobaric Heat Capacity

Several investigations on the isobaric heat capacity ($c_p$) of THF complement the data base of caloric properties. Isobaric heat capacity data cover the vapor and liquid phase at atmospheric pressure. A total of ten authors have contributed 129 data points. The available literature data is summarized in Table 10 including the AARD calculated with the present EOS.

**Table 10:** Data summary and average absolute relative deviations (AARD) of experimental data for isobaric heat capacity from the EOS

Reference	Year	No. of data	*T* (K)	*p* (MPa)	AARD (%)		
					Vap	Liq	Overall
Conti et al. [125]	1994	1	298.15	0.1	–	1.3	1.3
Conti et al. [72]	1998	1	298.15	0.1	–	1.4	1.4
Costas and Patterson [212]	1985	3	283–314	0.1	–	0.17	0.17
Diedrichs and Gmehling [213]	2006	30	180–326	0.1	–	1.3	1.3
Francesconi et al. [126]	2006	8	288–324	0.1	–	2.5	2.5
Giner et al. [209]	2007	2	298–314	0.1	–	0.25	0.25
Hossenlopp and Scott [24]	1981	11	328–501	0.025–0.2	0.31	–	0.31
Lebedev et al. [22]	1978	69	161–323	0.1	–	0.15	0.15
Nonay et al. [49]	2010	2	283–299	0.1	–	0.13	0.13
Rodríguez et al. [132]	1999	2	298–314	0.1	–	0.27	0.27

Clear outliers were not considered in the calculation of the AARD

**Fig. 15:**
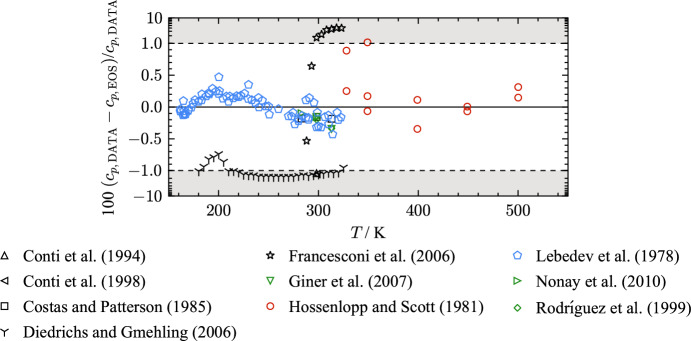
Percentage absolute deviation of isobaric heat-capacity data as a function of temperature from the present EOS

Figure 15 shows percentage deviations of isobaric heat capacity data from the present EOS as a function of temperature.

The most comprehensive data set is the study of Lebedev et al. [22] who investigated caloric properties of THF in the temperature range from 8 K to 322 K. The homogeneous liquid phase was studied in a temperature range between 161.5 K and 322.6 K at atmospheric pressure. For $c_p$ measurements, a vacuum adiabatic calorimetric cryostat was used. The authors assign an expanded uncertainty of 0.4 % ($k = 2$) to the experimental results. The present EOS describes this data set with an AARD of 0.15 % and a maximum deviation of 0.47 %. Figure 15 exhibits two clear outliers in the data set of Lebedev et al. [22] that cause the maximum deviation to be beyond the assigned uncertainty. Excluding these two data points, the data set is represented within the uncertainty. The data of Lebedev et al. [22] are in very good agreement with some other studies [49, 132, 209, 212] that used other measuring methods. The first three studies were published by authors from the same working group in a period of eleven years from 1999 to 2010 [49, 132, 209]. Rodríguez et al. [132] and Giner et al. [209] refer to the same publication [214] when introducing their measurement apparatus. Both studies carried out measurements at 298.15 K and 313.15 K. The experimental results of both studies seem reproducible according to Fig. 15. However, the authors do not provide any information regarding uncertainties. The AARD of the data from Rodríguez et al. [132] is 0.27 % with a maximum deviation 0.34 %. Isobaric heat capacity data of Giner et al. [209] are represented with an AARD of 0.25 % with a maximum deviation of 0.34 %. The latest study of the working group providing $c_p$ data for THF is from Nonay et al. [49]. At 283.15 K and 298.15 K and at atmospheric pressure, the authors performed measurements on a different experimental setup that is described by Góralski et al. [215]. Here, the authors provide an expanded uncertainty of 0.16 % ($k = 2$). With an AARD of 0.13 % and a maximum deviation of 0.16 %, the data of Nonay et al. [49] agree with the present EOS within their experimental uncertainty.

A study that is well in line with the prior four presented publications is from Costas and Patterson [212]. During the investigation of heat capacities of water and organic-solvent mixtures, the isobaric heat capacity of pure THF was measured in a temperature range from 283.15 K to 313.15 K at ambient pressure. The measurements were performed with a flow microcalorimeter, which is described in detail elsewhere [216]. Costas and Patterson [212] compared the results of $c_p$ measurements of two other fluids with literature values and suggest a standard uncertainty of 1 % ($k = 1$). Considering an AARD of 0.17 % and a maximum deviation of 0.185 %, it appears that this uncertainty is too pessimistic.

After the publication of Lebedev et al. [22], the most comprehensive data set is contributed by a study of Diedrichs and Gmehling [213]. The measurements were completed on a differential scanning calorimeter that operates with an expanded uncertainty of 5 % ($k = 2$) according to the manufacturer. The gathered data points deviate between 0.7 % and 1.7 % from the present EOS, and, therefore, are represented within their uncertainty.

Isobaric heat capacities in the gaseous phase were measured by Hossenlopp and Scott [24]. Measurements of the heat capacities were performed according to the constant flow method with the apparatus described by McCullough and Waddington [217]. McCullough and Waddington [217] assume that measurements can be performed with an uncertainty of 0.05 %. But due to possible systematic errors the authors suggest a standard uncertainty of 0.2 % ($k = 1$). For the data of Hossenlopp and Scott [24], an expanded uncertainty of 0.4 % ($k = 2$) is assigned. The AARD of the data set is 0.31 % and the maximum deviation 1.1 %. As shown in Fig. 15, two data points at 328 K and 349 K are not within the 0.4 % uncertainty. These data points are rated as outliers since they were measured at different isobars. Without those two outliers, the data set is represented within 0.35 %.

The present EOS has an uncertainty of 0.4 % for $c_p$ in the liquid phase at atmospheric pressure in a temperature range of 160 K to 320 K. In the vapor phase, the uncertainty is estimated to be 0.2 % in a temperature range of 328 K to 500 K at pressures up to 0.1 MPa. Uncertainty estimates exceeding these ranges cannot be specified.

## Extrapolation Behavior

Investigating the extrapolation behavior provides important information about the quality of an equation of state. When used in mixture models, pure-fluid EOS are often evaluated at state points outside the range of validity of the pure-fluid equations. Thus, reasonable extrapolation behavior is crucial for mixture models. Courses of constant property lines have to show appropriate characteristics at extreme temperature and pressure, as well as smooth transitions when approaching the critical region. Based on various criteria, the physical behavior of the new equation of state for THF is reviewed in terms of multiple thermal and caloric properties.

**Fig. 16:**
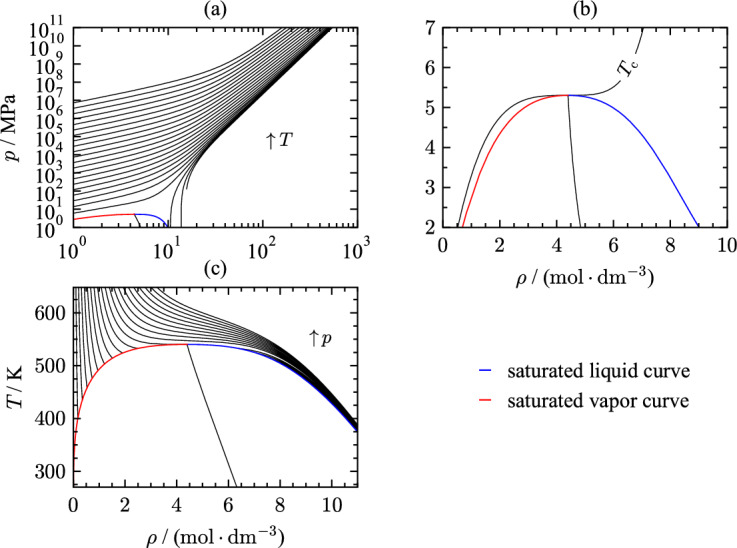
$p,\rho$-diagram on a double logarithmic scale along isotherms up to 10^6^ K (a), $p,\rho$-diagram along the the critical isotherms (b), and $T,\rho$-diagram along isobars (c) calculated with the present EOS

Figure 16 presents the extrapolation behavior of thermal properties of THF. The projection of extreme conditions is illustrated in Fig. 16(a) as a double-logarithmic $p,\rho$ diagram along isotherms of up to 10$^{6}$ K. The isotherms show a smooth transition between phases without exhibiting intersections or sudden curvature changes at high pressures. Figure 16(b) displays the behavior of the present EOS in the critical region in terms of thermal properties. The critical point of pure fluids is defined by the saddle point along the critical temperature as a function of density [$(\partial p/\partial \rho )_{T_{\text{c}}}=(\partial ^2 p/\partial \rho ^2)_{T_{\text{c}}}=0$]. The present EOS aligns with these criteria. Another measure for correct physical behavior is the rectilinear diameter that is defined as the arithmetic mean of the saturated vapor and liquid densities at the same temperature, $\rho _{\text{RD}}~=~(\rho ''+\rho ')/2$. The rectilinear diameter is included in all diagrams in Fig. 16 and should behave linearly in the vicinity of the critical point, according to Zollweg and Mullholland [218].

**Fig. 17:**
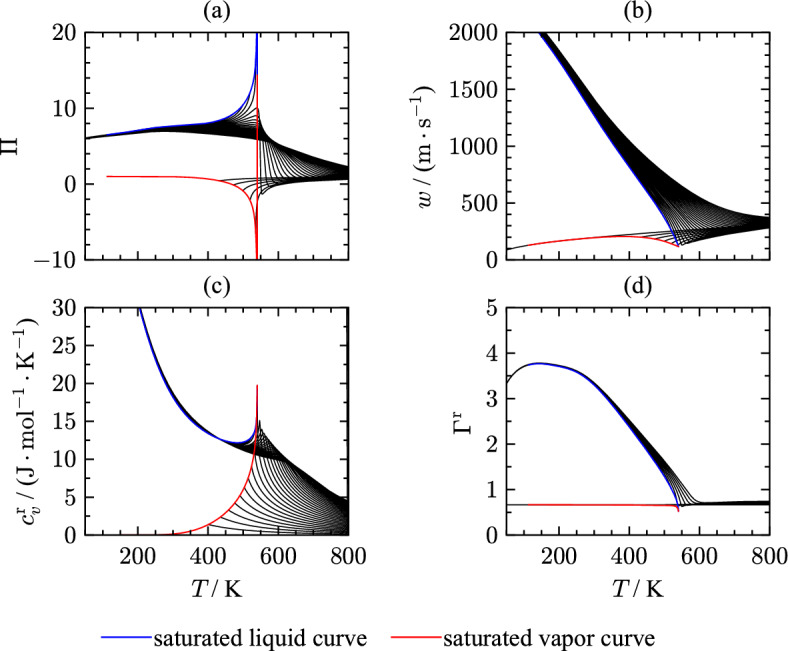
Phase identification parameter (a), speed of sound (b), residual isochoric heat capacity (c), and residual Grüneisen parameter (d) along isobars as a function of temperature from 50 K to 800 K

Figure 17 shows several thermodynamic properties as a function of temperature that have been considered during the development of the EOS.

The phase identification parameter (PIP) [219] combines first and second order pressure derivatives with respect to temperature and density. This parameter identifies the phase without needing an iterative solution and is formulated as:

17 $$\begin{aligned} \Pi = 2 - \rho \left[ \frac{\left( \partial ^2 p/\partial \rho \partial T\right) }{\left( \partial p / \partial T \right) _\rho } - \frac{\left( \partial ^2 p/\partial \rho ^2\right) _T}{\left( \partial p / \partial \rho \right) _T}\right] . \end{aligned}$$

The course of saturation lines should be smooth without sudden curvature changes ending in a maximum (saturated liquid) and minimum (saturated vapor) at the critical temperature. Surpassing the critical temperature, the isobars should converge with a change in curvature. Isobars above the critical pressure should show a curvature change when transitioning from liquid to supercritical states. The present EOS fulfills all these criteria. Speed of sound (Fig. 17(b)) includes the pressure derivative $(\partial p / \partial T)_\rho$ which is infinite at the critical point. In terms of sound speed, the partial pressure derivative leads to a local minimum of speed of sound at the critical point. The isobars in the liquid phase have all negative derivatives, whereas isobars in the gaseous and supercritical regions show positive slope and negative curvature. To assess the residual part of the Helmholtz energy, the residual isochoric heat capacity is used as a function of temperature as displayed in Fig. 17(c). This plot underlines the course of the phase boundaries as well as the behavior in the critical region. Before approaching the critical point, the vapor and liquid saturation lines should cross once and have positive slope and curvature. The saturation lines meet at the critical temperature forming a pronounced maximum in $c_v^{\text{r}}$. Considering the ideal-gas behavior, the residual isochoric heat capacity should converge to its ideal-gas limiting value of zero at high temperatures.

An additional parameter consisting of partial derivatives is the residual Grüneisen parameter [220] that also allows assessing the extrapolation behavior of EOS:

18 $$\begin{aligned} \Gamma ^{\text{r}} = c_v^{\text{r}}\frac{(\partial p / \partial T)_\rho }{\rho }. \end{aligned}$$

This parameter combines thermal and caloric properties. Therefore, it is of special interest for validating the extrapolation behavior of the EOS. Due to the partial derivative of pressure with respect to the temperature and the dependence on the isochoric heat capacity, the shape of the Grüneisen parameter as a function of temperature is comparable to the behavior of speed of sound over temperature. For evaluating the EOS, special attention was placed on the residual Grüneisen parameter at low temperatures to yield smooth extrapolation behavior beyond the triple point. The course of isobars in the liquid phase in Fig. 17(d) is smooth with negative curvature. The saturation lines meet at the critical point where the residual Grüneisen parameter demonstrates a minimum. Therewith, the present EOS shows correct extrapolation behavior in all displayed properties in Fig. 17.

**Fig. 18:**
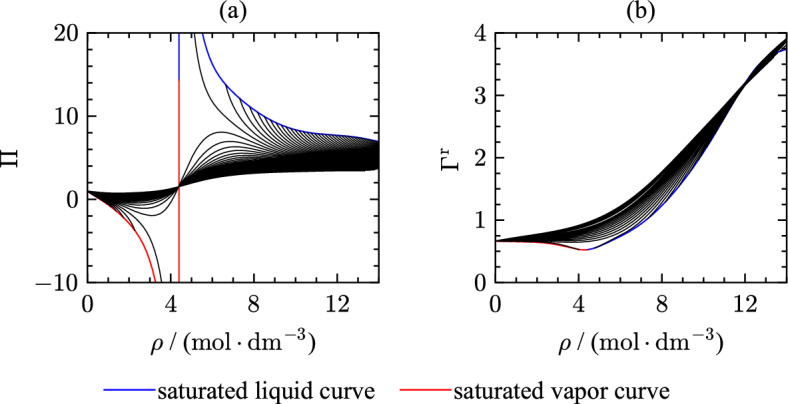
Phase identification parameter (a) and residual Grüneisen parameter (b) along isotherms as a function of density

Figure 18 illustrates the PIP and Grüneisen parameter as a function of density along isotherms. Isotherms in the $\Pi ,\rho$ plot (Fig. 18(a)) should cross at the critical density ($\rho _{\text{c}} = 4.22$ ${\text{mol}}{\cdot}{\text{dm}}^{-3}$) and change curvature. The formed minima and maxima should be more pronounced as isotherms approach the critical temperature. The course of the saturated liquid line should be smooth over the entire density range. The residual Grüneisen parameter as a function of density is informative about the behavior at high densities. Approaching high densities, the isotherms should cross and diverge again. While the present EOS agrees with criteria for the PIP as a function of density, isotherms of the residual Grüneisen parameter show questionable behavior.

**Fig. 19:**
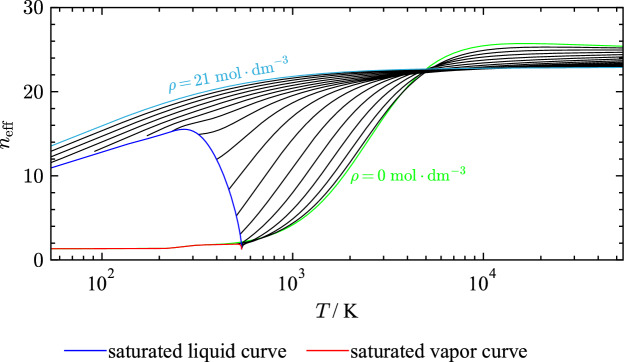
Values of $n_{\text{eff}}$ along isochores as a function of temperature. The green solid line corresponds to values of $n_{\text{eff}}$ in the ideal-gas limit

In ongoing efforts to link thermodynamic and transport properties, Bell [221] introduced the effective hardness of interaction ($n_{\text{eff}}$) for the evaluation of equations of state. Originating from density scaling, this parameter has a strong connection to thermodynamic theory and can be understood as the effective repulsiveness of the interaction between molecules [221]. The $n_{\text{eff}}$ is defined as:

19 $$\begin{aligned} n_{\text{eff}} = 3\frac{\rho }{T}\left( \frac{\partial T}{\partial \rho } \right) _{s^{\text{r}}} = 3R\frac{\left( \frac{\partial (p^{\text{r}}/R) }{ \partial T}\right) _\rho }{\rho c_v^{\text{r}}}, \end{aligned}$$

where $s^{\text{r}}$ denotes the residual entropy and $p^{\text{r}}$ the residual pressure. Bell [221] studied values of $n_{\text{eff}}$ at the ideal-gas limit, and latter extended its application to the entire fluid phase [222]. Values of $n_{\text{eff}}$ calculated with the present EOS for THF as a function of temperature along isochores are shown in Fig. 19. In the ideal-gas limit ($\rho = 0$ ${\text{mol}}{\cdot}{\text{dm}}^{-3}$), values of $n_{\text{eff}}$ should increase smoothly towards a global maximum. Values of the neff along the ideal-gas isochore should always be positive; negative values indicate negative values of $c_v^{\text{r}}$ in the gaseous phase. While the EOS for THF fulfills this condition, the curvature below the critical temperature is not always positive. High density isochores should have negative curvature with positive slope, cross the ideal-gas isochore, and converge to an infinite temperature limit. The liquid saturation curve should come to a maximum and then decrease smoothly to a global minimum at the critical point. The present EOS shows proper extrapolation behavior in terms of the $n_{\text{eff}}$.

**Fig. 20:**
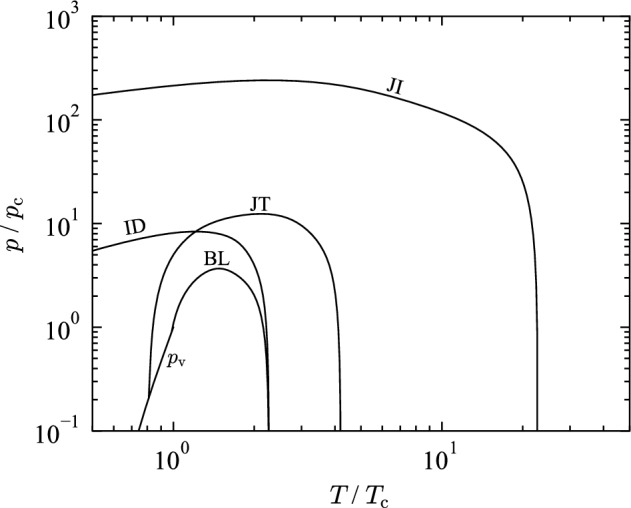
Ideal curves: Joule inversion curve (JI), Joule–Thomson inversion curve (JT), ideal curve (ID), Boyle curve (BL), vapor pressure curve ($p_{\text{v}}$)

Another criterion for evaluating an EOS are so-called “ideal-curves”, see Fig. 20. Along ideal curves, any property of the real fluid corresponds to the hypotheoretical ideal gas for the same temperature and density. Typically, they are defined in terms of the compressibility factor *Z* and its derivatives [223]. Generally, ideal curves are examined with the ideal curve [where $Z=1$], the Boyle curve [where $\left( \partial Z/\partial \rho \right) _T=0$], the Joule–Thomson inversion curve [where $\left( \partial Z/\partial T\right) _p=0$], and the Joule inversion curve [where $\left( \partial Z/\partial T\right) _\rho =0$]. Ideal curves calculated with the present EOS exhibit good extrapolation without sudden changes in slope or curvature over a broad temperature range.

**Fig. 21:**
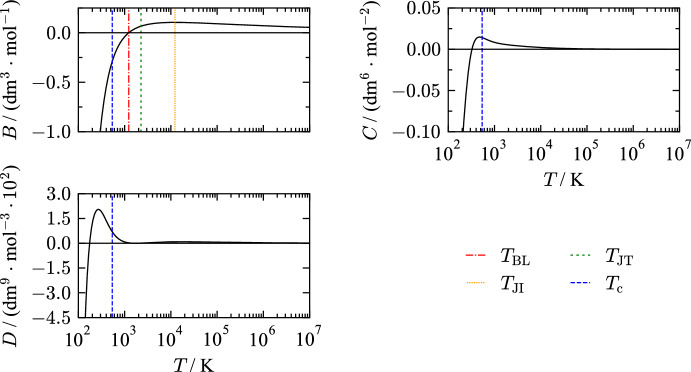
Second (*B*), third (*C*), fourth (*D*) virial coefficient calculated with the present EOS

The virial coefficients of THF are shown in Fig. 21. Virial coefficients can be derived from *pvT* and speed of sound measurements in the gaseous and supercritical phase and, thus, demonstrate the extrapolation behavior of equations of state. Typical characteristics for any virial coefficient are a zero-crossing, followed by a global maximum, and a convergence towards zero at high temperatures. Approaching low temperatures, virial coefficients should result in large negative values, accounting for an attraction-dominated interaction between the molecules. All virial coefficients fulfill the mentioned criteria. Maxima are usually formed at characteristic temperatures that are connected to values of ideal curves at zero pressure or density. The second virial coefficient shows a maximum at the Joule-Thompson temperature and crosses the zero line at the Boyle temperature. The third virial coefficient has a maximum near the critical temperature followed by converging towards zero at high temperatures. In a study on the Lennard–Jones fluid, Thol *et* *al*. [224] found that a plateau forms at higher temperatures that is not present in this EOS. In addition to the global maximum, the correct physical behavior of fourth virial coefficient *D* results in a second smaller maximum on a temperature plot that is also reflected by the present EOS.

Overall, the discussed thermodynamic properties demonstrate good extrapolation capabilities of the present EOS for THF.

## Conclusion

A fundamental equation of state in terms of the Helmholtz energy for THF is presented. The formulation consists of an ideal-gas contribution with four Planck–Einstein terms and a residual part comprising five monomial, five exponential, and five Gaussian bell-shaped terms. Combinations of its derivatives allow for the calculation of all thermodynamic properties. The range of validity covers a temperature range from the triple-point temperature ($T_{\text{tr}}= 1$ 64.15 K) up to 550 K with pressures up to 600 MPa. Additionally, ancillary equations for vapor pressure $p_{\text{v}}$, saturated liquid density $\rho '$, and saturated vapor density $\rho ''$ were developed for fast calculation of saturation state points.

The estimated uncertainty of calculated liquid densities with the present EOS is 0.015 % in a temperature range from 275 K to 320 K at ambient pressure. For temperatures below 275 K at atmospheric pressure, we report an uncertainty of 0.7 %. Liquid densities above 0.1 MPa have an estimated uncertainty of 0.2 % between 278 K and 450 K. The uncertainty of the EOS in speed of sound is estimated to be 0.03 % at atmospheric pressure for temperatures from 278 K to 320 K. The uncertainty increases to 1.5 % for temperatures down to 240 K. At atmospheric pressure, calculations of the isobaric heat capacity between 160 K and 500 K have an estimated uncertainty of 0.4 % in the liquid phase and 0.2 % in the vapor phase. Beyond ambient pressure conditions, no uncertainty estimation is possible regarding speed of sound and isobaric heat capacity. Saturation properties were assessed in terms of vapor pressure and heat of vaporization. Vapor pressure is represented with an uncertainty of 0.05 % for temperatures up to 375 K and 3 % above 375 K. The expected uncertainty in heat of vaporization is 0.8 % for temperatures between 300 K and 340 K. The present EOS for THF will be available in future releases of REFPROP [225], TREND [226], and CoolProp [227]. Table 11 in Appendix provides test values for computer implementation calculated with TREND [226].

### Supplementary Information

Below is the link to the electronic supplementary material.

Supplementary file1 (ZIP 278 kb)

## Data Availability

There are no new data sets available. The applied data are taken from literature and marked with the corresponding references.

## Appendix A: Validation Data for Implementation

See Table 11.

**Table 11:** Test values in the single-phase region for computer implementation

*T* (K)	$\rho$ (${\text{mol}}\cdot{\text{m}}^{-3}$)	*p* (MPa)	$c_p$ (${\text{J}}{\cdot}{\text{mol}}^{-1}{\cdot}{\text{K}}^{-1}$)	*w* (${\text{m}}{\cdot}{\text{s}}^{-1}$)
270	$10^{-4}$	0.0 000 002 245	67.962 189 060	188.343 575 267
350	40	0.1 130 755 690	93.540 905 193	205.594 935 289
450	10 000	12.357 974 600	167.23 826 646	739.195 761 440
550	5000	6.1 720 378 363	763.57 251 979	139.994 309 340

*T* (K)	$\rho$ (${{\text{mol}}\cdot{\text{m}}^{-3}}$)	*h* (${\text{J}}{\cdot}{\text{mol}}^{-1}$)	*s* (${\text{J}}{\cdot}{\text{mol}}^{-1}{\cdot}{\text{K}}^{-1}$)	*a* (${\text{J}}{\cdot}{\text{mol}}^{-1}$)
270	$10^{-4}$	24 850.336 600	179.51 672 775	− 25 864.084 532
350	40	30 997.192 355	90.484 710 729	− 3499.345 627
450	10 000	17 240.429 690	40.905 432 334	− 2402.812 320
550	5000	40 236.271 714	87.703 653 509	− 9235.145 283
