# Supplementary material for: Fundamental Equation of State for Fluid Tetrahydrofuran
Source: Int J Thermophys. 2023 Oct 9;44(10):153. doi: 10.1007/s10765-023-03258-3 (PMC10562338; doi:10.1007/s10765-023-03258-3)
Supplement: Supplementary file 1 — Supplementary file1 (ZIP 278 kb) [file 10765_2023_3258_MOESM1_ESM.zip › Supplementary/IntJThermophys_THF_supplementary.pdf]

Supplementary Material for "Fundamental Equation of State for Fluid Tetrahydrofuran"

Felix Fiedler[1*], Joel Karog[1], Eric W. Lemmon[2], Monika Thol[1]

[1]Lehrstuhl für Thermodynamik, Ruhr-Universität Bochum, Universitätsstraße 150, Bochum, 44801, Germany.
[2]Applied Chemicals and Materials Division, National Institute of Standards and Technology, 325 Broadway, Boulder, 803005, Colorado, USA.

*Corresponding author(s). E-mail(s): f.fiedler@thermo.rub.de;

Keywords: Tetrahydrofuran, THF, thermodynamic properties, equation of state, Helmholtz energy

This supplemental material provides a Table of all selected homogeneous density publications including average absolute relative deviations (AARD) calculated with the present equation of state (EOS), and deviation plots of all considered density data in temperature and pressure intervals.

1 Homogeneous Density AARD Table

Table 1: Data summary and average absolute relative deviations (AARD) of experimental data for homogeneous densities in the liquid phase from the EOS. Clear outliers were not considered in the calculation of the AARD.

Reference	Year	No. of Data	T / K	p / MPa	AARD / %
Acevedo et al. [1]	2001	1	298.15	0.101325	0.012
Afanasyev and Zyatkova [2]	1996	3	283−314	0.101325	0.19
Almasi [3]	2018	4	298−314	0.101325	0.014
Amigo et al. [4]	1993	1	298.15	0.101325	0.006

Continued on next page

Table 1 continued

Reference	Year	No. of Data	T / K	p / MPa	AARD / %
Aminabhavi and Gopalakrishna [5]	1995	1	298.15	0.101325	0.033
Aminabhavi and Patil [6]	1998	3	298−309	0.101325	2.3
Aminabhavi et al. [7]	1989	3	298−319	0.101325	0.77
Anantaraman [8]	1986	1	278.15	0.101325	0.034
Aralaguppi et al. [9]	1996	3	298−309	0.101325	0.090
Back and Woolf [10]	1998	45	278−324	0.1−300	0.056
Bandrés et al. [11]	2007	1	298.15	0.101325	0.001
Bandrés et al. [12]	2008	2	298−329	0.101325	0.99
Bandrés et al. [13]	2008	1	298.15	0.101325	0.001
Bardavid et al. [14]	1996	1	298.15	0.101325	0.022
Belandria et al. [15]	2009	9	293−334	0.101325	0.87
Bernazzani et al. [16]	2002	1	298.15	0.101325	0.003
Bhattacharyya et al. [17]	1965	1	298.14	0.101325	0.23
Bourns and Nicholls [18]	1948	1	293.14	0.101325	0.058
Brocos et al. [19]	1996	3	288−309	0.101325	0.009
Carvajal et al. [20]	1965	10	203−299	0.101325	0.61
Chen et al. [21]	2015	7	293−324	0.101325	0.041
Choudhury et al. [22]	2003	5	303−323	0.101325	0.22
Comelli and Francesconi [23]	1991	10	290−304	0.101325	0.19
Comelli and Francesconi [24]	1995	1	298.15	0.101325	0.078
Conti et al. [25]	1994	2	298.15	0.101325	0.013
Conti et al. [26]	1998	1	298.15	0.101325	0.021
Das and Roy [27]	2006	3	298−319	0.101325	0.11
Das et al. [28]	1994	3	298−318	0.101325	0.14
Deshpande et al. [29]	1971	1	298.14	0.101325	0.27
Dhaduk et al. [30]	2015	4	298−313	0.101325	0.030
Fattahi and Iloukhani [31]	2010	3	288−309	0.101325	0.005
Fenclová and Dohnal [32]	1991	1	298.15	0.101325	0.001
Francesconi and Comelli [33]	1992	1	298.15	0.101325	0.038
Francesconi and Comelli [34]	1994	1	298.15	0.101325	0.048
Francesconi and Comelli [35]	1995	1	298.15	0.101325	0.030
Francesconi and Comelli [36]	1995	1	298.15	0.101325	0.031
Francesconi et al. [37]	1992	1	298.15	0.101325	0.019
Francesconi et al. [38]	1993	1	298.15	0.101325	0.021
Francesconi et al. [39]	2006	2	298−309	0.101325	0.009
Gadžurić et al. [40]	2012	5	293−314	0.101325	0.13
Gascón et al. [41]	1999	2	298−314	0.101325	0.006
Gascón et al. [42]	2000	1	298.15	0.101325	0.001
Geppert-Rybczynska and Sitarek [43]	2014	1	298.15	0.1	0.006
Gill et al. [44]	1993	1	298.00	0.101325	0.059
Giner et al. [45]	2006	2	298−314	0.101325	0.004
Giner et al. [46]	2007	2	298−314	0.101325	0.003
Giner et al. [47]	2007	3	298−329	0.101325	0.015

Continued on next page

Table 1 continued

Reference	Year	No. of Data	T / K	p / MPa	AARD / %
Govender et al. [48]	1996	50	288−329	0.1−8.0	0.56
Grolier et al. [49]	1982	1	298.14	0.101325	0.013
Gupta et al. [50]	2003	1	293.00	0.101325	0.23
Hnedkovsky and Cibulka [51]	1986	1	298.14	0.101325	0.020
Holland and Smyth [52]	1955	3	274−314	0.101325	0.14
Ijardar and Malek [53]	2014	7	293−324	0.101325	0.090
Iloukhani et al. [54]	2005	1	298.15	0.101325	0.007
Inglese et al. [55]	1983	3	298−319	0.101325	0.11
Ivanov [56]	2011	5	278−319	0.101325	0.008
Ivanov [57]	2014	9	278−319	0.101325	0.006
Jagodzinski and Petrucci [58]	1974	1	298.14	0.101325	0.013
Jain et al. [59]	1979	1	303.14	0.101325	0.30
Jatkar and Deshpande [60]	1960	7	298−329	0.101325	0.14
Jha et al. [61]	2003	6	298−323	0.101325	0.21
Kalali et al. [62]	1990	1	293.14	0.101325	0.19
Katuno [63]	1948	1	293.14	0.101325	0.10
Kinart et al. [64]	2002	5	291−309	0.101325	0.004
Kiyohara et al. [65]	1979	1	298.14	0.101325	0.005
Klages and Möhler [66]	1948	4	293−354	0.101325	0.19
Kneževic-Stevanovic et al. [67]	2013	8	288−324	0.101325	0.74
Kneževic-Stevanovic et al. [68]	2014	2	298.15	0.101325	0.041
Komatsu and Masumoto [69]	1930	1	298.14	0.101325	1.7
Koroesi and Kovats [70]	1981	1	293.14	0.101325	0.10
Ku et al. [71]	2008	3	288−309	0.101325	0.031
Kumar [72]	2000	6	288−339	0.101325	1.8
Kushare et al. [73]	2006	1	298.15	0.101325	0.006
Lafuente et al. [74]	2001	1	298.15	0.101325	0.009
Lejcek et al. [75]	1975	1	298.14	0.101325	0.044
Lepori and Matteoli [76]	1986	1	298.14	0.101325	0.020
Letcher and Domanska [77]	1994	1	298.15	0.101325	0.030
Letcher and Domanska [78]	1994	1	298.15	0.101325	0.078
Loras et al. [79]	1999	1	298.15	0.101325	0.011
Loras et al. [80]	2002	2	293−299	0.101325	0.007
Marczak et al. [81]	2008	5	297−314	0.101325	0.023
Mariano et al. [82]	2000	3	283−314	0.101325	0.050
Matous et al. [83]	1972	1	298.14	0.101325	0.021
Matsuda et al. [84]	2011	1	298.15	0.101325	0.004
Metz and Glines [85]	1967	1	298.14	0.101325	0.11
Muhuri et al. [86]	1996	3	298−319	0.101325	0.14
Nain [87]	2006	9	278−319	0.101325	0.14
Nain and Droliya [88]	2017	6	293−319	0.101325	0.13
Naorem and Suri [89]	1989	1	298.14	0.101325	0.010
Nayak et al. [90]	2003	3	298−309	0.101325	0.082
Nayak et al. [91]	2004	3	303−324	0.101325	0.025
Nicolas et al. [92]	1980	7	223−293	0.101325	0.32

Continued on next page

Table 1 continued

Reference	Year	No. of Data	T / K	p / MPa	AARD / %
Nikolic et al. [93]	2005	4	303−319	0.101325	0.051
Nikolic et al. [94]	2006	4	303−319	0.101325	0.051
Nonay et al. [95]	2010	3	283−314	0.101325	0.017
Oshmyansky et al. [96]	1986	2	298−319	0.101325	0.16
Oswal et al. [97]	2005	1	303.15	0.101325	0.024
Oswal et al. [98]	2005	1	303.15	0.101325	0.024
Oswal et al. [99]	2010	3	303−324	0.101325	0.20
Ottani et al. [100]	2002	6	297−308	0.101325	0.015
Ottani et al. [101]	2003	3	288−314	0.101325	0.018
Palani and Geetha [102]	2009	3	303−313	0.101325	0.19
Palani et al. [103]	2009	2	308−313	0.101325	0.12
Pandiyan et al. [104]	2011	3	303−324	0.101325	0.058
Peralta et al. [105]	2003	1	298.15	0.101325	0.066
Peralta et al. [106]	2005	1	298.15	0.101325	0.004
Pérez et al. [107]	2003	3	283−314	0.101325	0.038
Piñeiro et al. [108]	2002	11	293−304	0.101325	0.006
Postigo et al. [109]	2003	3	283−314	0.101325	0.050
Prasad et al. [110]	2005	1	293.15	0.101325	0.19
Qun-Fang et al. [111]	1997	1	293.15	0.101325	0.17
Ramkumar and Kudchadker [112]	1989	5	278−299	0.101325	0.024
Rathnam [113]	1988	1	303.14	0.101325	0.26
Rathnam et al. [114]	2013	3	303−314	0.101325	0.22
Rathnam et al. [115]	2013	4	298−314	0.101325	0.18
Rodnikova et al. [116]	2011	5	293−334	0.101325	0.008
Rodríguez et al. [117]	1996	1	298.15	0.101325	0.009
Rodríguez et al. [118]	1997	1	298.15	0.101325	0.009
Rodríguez et al. [119]	1999	2	298−314	0.101325	0.008
Rodríguez et al. [120]	2000	1	298.15	0.101325	0.009
Rodríguez et al. [121]	2006	1	298.15	0.101325	0.009
Roy et al. [122]	2001	5	298−318	0.101325	0.10
Saleh et al. [123]	2002	5	303−324	0.101325	0.53
Schedemann [124]	2009	459	283−443	0.3−130	0.12
Schornack and Eckert [125]	1970	14	303−324	0.1−517	1.5
Segura et al. [126]	2003	1	298.15	0.101325	0.032
Shelar et al. [127]	2016	4	298−313	0.101325	0.26
Sinha and Roy [128]	2006	3	298−319	0.101325	0.12
Sinha and Roy [129]	2006	3	303−324	0.101325	0.23
Sinha et al. [130]	2013	3	298−319	0.101325	0.12
Smyth and Walls [131]	1932	1	293.14	0.101325	1.0
Solimo and Gomez Marigliano [132]	1993	1	303.15	0.101325	0.15
Surendranath et al. [133]	1992	1	303.15	0.101325	0.30
Suri and Naorem [134]	1987	1	298.14	0.101325	0.010
Taniewska-Osinska et al. [135]	1993	1	298.15	0.101325	0.012
Torres et al. [136]	2008	4	288−304	0.101325	0.006
Vaid et al. [137]	2015	7	293−324	0.101325	0.15

Continued on next page

Table 1 continued

Reference	Year	No. of Data	T / K	p / MPa	AARD / %
Valén et al. [138]	2002	3	283−314	0.101325	0.004
Valén et al. [139]	2002	3	283−314	0.101325	0.004
Valen et al. [140]	2003	3	283−314	0.101325	0.004
Vercher et al. [141]	2011	5	278−319	0.1	0.007
Villares et al. [142]	2004	1	298.15	0.101325	0.009
Vittal Prasad et al. [143]	2004	1	298.15	0.101325	0.81
Wang et al. [144]	2002	1	298.15	0.101325	0.000
Wang et al. [145]	2003	1	298.15	0.101325	0.009
Wankhede et al. [146]	2008	3	288−309	0.101325	0.006
Wankhede et al. [147]	2010	3	288−304	0.101325	0.22
Weissler [148]	1949	1	303.13	0.101325	0.23
Whitehead et al. [149]	1951	1	293.14	0.101325	0.17
Zafarani-Moattar & Majdan-Cegincara [150]	2007	1	298.15	0.101325	0.000
Zikmundova et al. [151]	1990	1	293.14	0.101325	0.60
Živkovic et al. [152]	2014	8	288−324	0.101325	0.049
Zurita et al. [153]	1992	2	298−309	0.101325	0.059

2 Homogeneous Density Deviation Plots

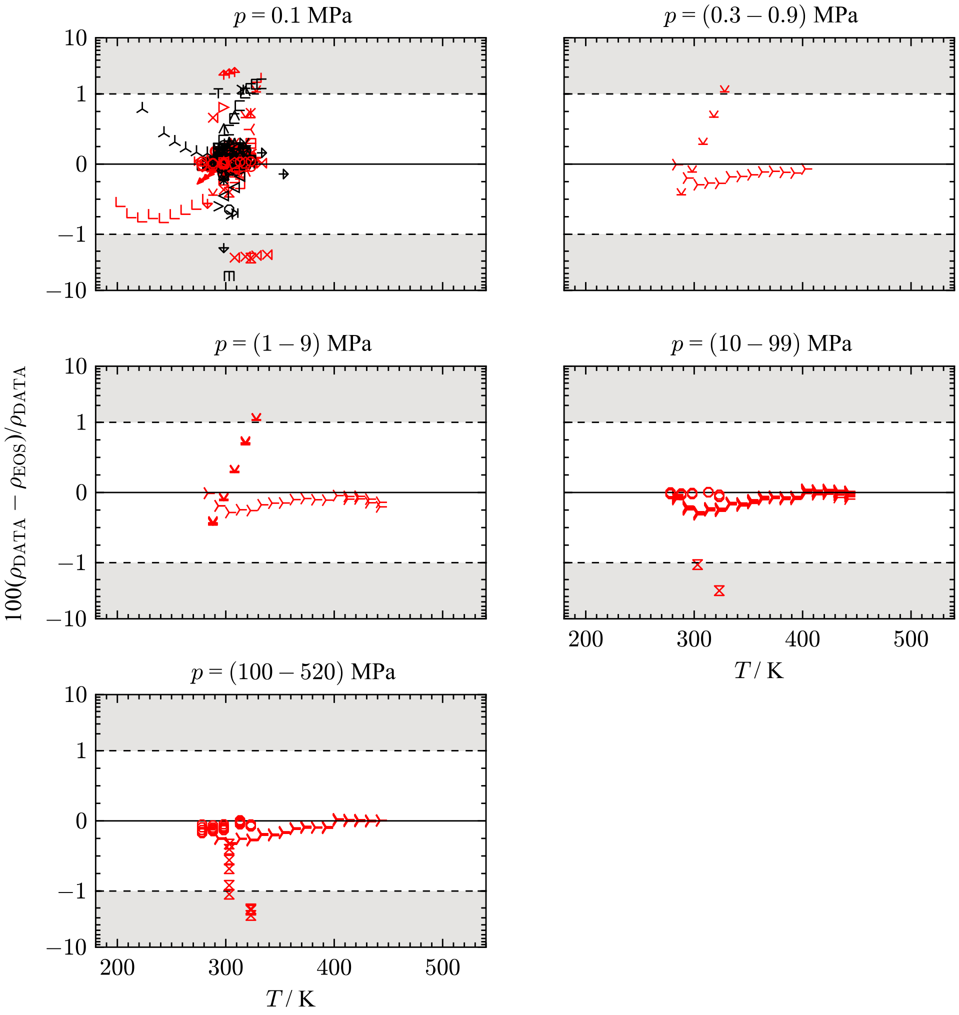

Fig. 1: Percentage relative deviation of density measurements as a function of temperature separated into pressure ranges. See Fig. 3 for symbol referencing.

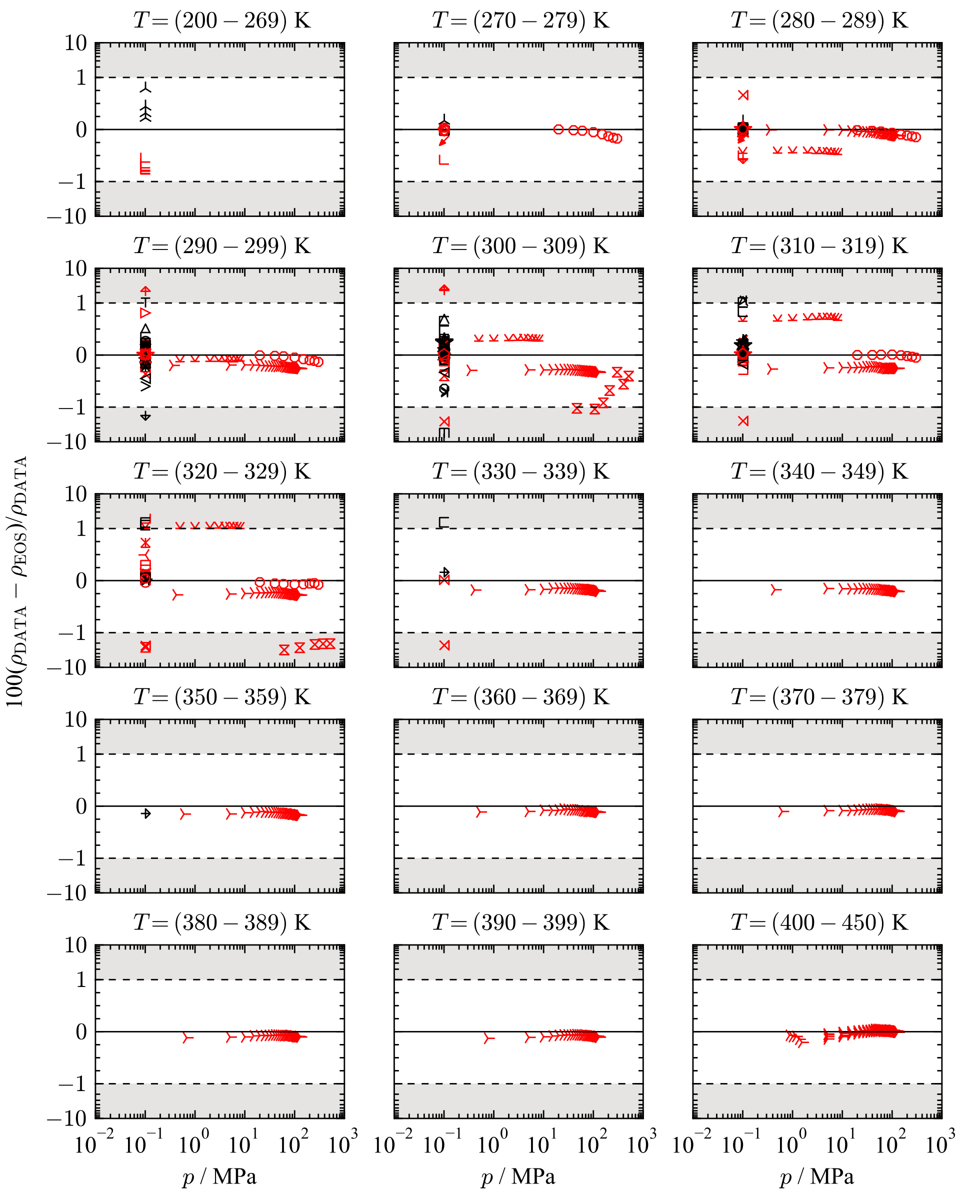

Fig. 2: Percentage relative deviation of density measurements as a function of pressure separated into temperature ranges. See Fig. 3 for symbol referencing.

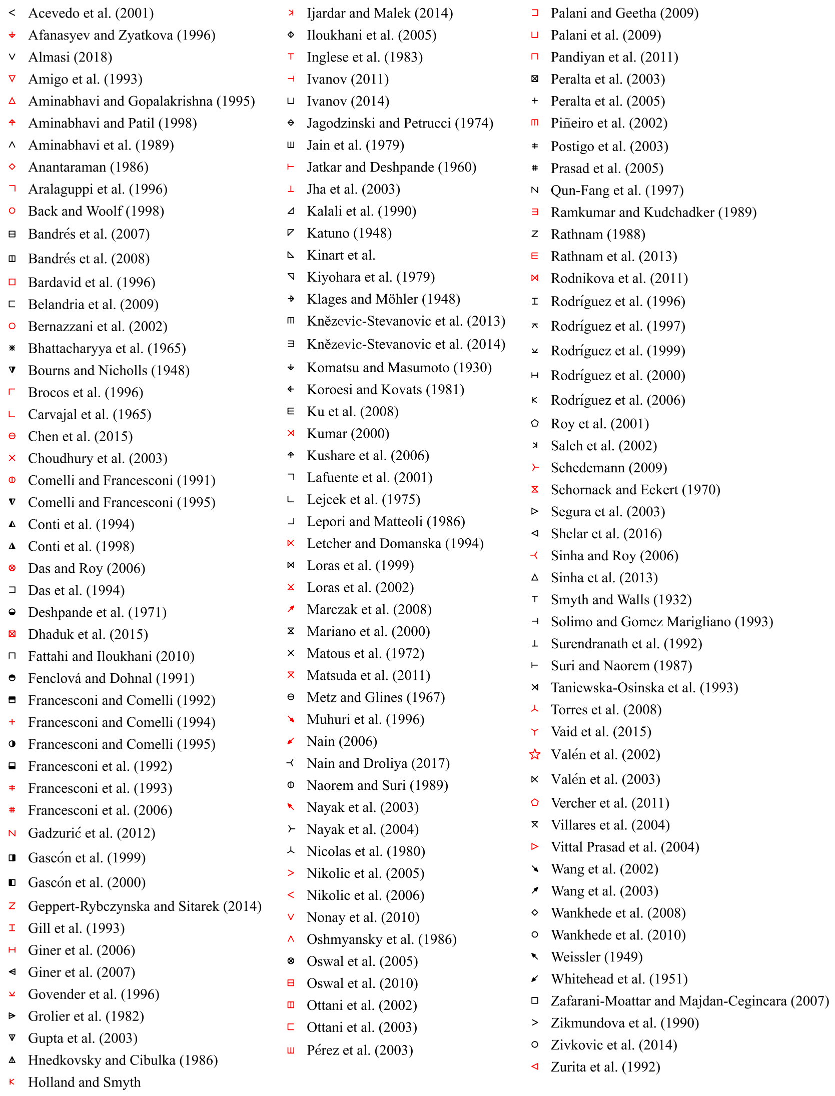

Fig. 3: Legend for Figures 1 and 2.

References

[1] Acevedo, I.L., Pedrosa, G.C., Arancibia, E.L., Katz, M. J. Sol. Chem. **30**(11), 1029–1037 (2001) https://doi.org/10.1023/A:1013355411329

[2] Afanasyev, V., Zyatkova, L. J. Chem. Eng. Data **41**(6), 1315–1318 (1996) https://doi.org/10.1021/je960003k

[3] Almasi, M. Phys. Chem. Res. **6**(3), 599–612 (2018) https://doi.org/10.22036/pcr.2018.126180.1476

[4] Amigo, A., Bravo, R., Pintos, M. J. Chem. Eng. Data **38**(1), 141–142 (1993) https://doi.org/10.1021/je00009a035

[5] Aminabhavi, T.M., Gopalakrishna, B. J. Chem. Eng. Data **40**(4), 856–861 (1995) https://doi.org/10.1021/je00020a026

[6] Aminabhavi, T.M., Patil, V.B. J. Chem. Eng. Data **43**(4), 497–503 (1998) https://doi.org/10.1021/je980031y

[7] Aminabhavi, T.M., Manjeshwar, L.S., Halligudi, S.B., Balundgi, R.H. Indian J. Chem. **28**(3), 217–221 (1989)

[8] Anantaraman, A.V. Can. J. Chem. **64**(1), 46–50 (1986) https://doi.org/10.1139/v86-010

[9] Aralaguppi, M.I., Jadar, C.V., Aminabhavi, T.M. J. Chem. Eng. Data **41**(6), 1307–1310 (1996) https://doi.org/10.1021/je960133t

[10] Back, P.J., Woolf, L.A. J. Chem. Thermodyn. **30**(3), 353–364 (1998) https://doi.org/10.1006/jcht.1997.0310

[11] Bandrés, I., Giner, I., Pera, G., Giner, B., Lafuente, C. Fluid Phase Equilib. **257**(1), 70–77 (2007) https://doi.org/10.1016/j.fluid.2007.05.013

[12] Bandrés, I., Giner, B., Villares, A., Artigas, H., Lafuente, C. J. Mol. Liq. **139**(1-3), 138–142 (2008) https://doi.org/10.1016/j.molliq.2007.12.002

[13] Bandrés, I., Giner, B., López, M.C., Artigas, H., Lafuente, C. J. Chem. Thermodyn. **40**(8), 1253–1260 (2008) https://doi.org/10.1016/j.jct.2008.03.017

[14] Bardavid, S.M., Pedrosa, G.C., Katz, M., Postigo, M.A., Garca, P. J. Sol. Chem. **25**(11), 1125–1135 (1996) https://doi.org/10.1007/BF00972927

[15] Belandria, V., Mohammadi, A.H., Richon, D. J. Chem. Thermodyn. **41**(12), 1382–1386 (2009) https://doi.org/10.1016/j.jct.2009.06.014

[16] Bernazzani, L., Mollica, V., Tiné, M.R. Fluid Phase Equilib. **203**(1-2), 15–29

(2002) https://doi.org/10.1016/S0378-3812(02)00169-3

[17] Bhattacharyya, D.N., Lee, C.L., Smid, J., Szwarc, M. J. Phys. Chem. **69**(2), 608–611 (1965) https://doi.org/10.1021/j100886a042

[18] Bourns, A.N., Nicholls, R.V.V. Can. J. Res. **26**(Sect B 1), 81–88 (1948) https://doi.org/10.1139/cjr48b-010

[19] Brocos, P., Amigo, A., Pintos, M., Calvo, E., Bravo, R. Thermochim. Acta **286**(2), 297–306 (1996) https://doi.org/10.1016/0040-6031(96)02956-5

[20] Carvajal, C., Tölle, K.J., Smid, J., Szwarc, M. J. Am. Chem. Soc. **87**(24), 5548–5553 (1965) https://doi.org/10.1021/ja00952a005

[21] Chen, F., Yang, Z., Chen, Z., Hu, J., Chen, C., Cai, J. J. Mol. Liq. **209**, 683–692 (2015) https://doi.org/10.1016/j.molliq.2015.06.041

[22] Choudhury, A., Jha, A., Roy, M.N. J. Ind. Chem. Soc. **80**(6), 632–636 (2003) https://doi.org/10.5281/ZENODO.5839787

[23] Comelli, F., Francesconi, R. J. Chem. Eng. Data **36**(4), 382–383 (1991) https://doi.org/10.1021/je00004a011

[24] Comelli, F., Francesconi, R. J. Chem. Eng. Data **40**(1), 28–30 (1995) https://doi.org/10.1021/je00017a007

[25] Conti, G., Gianni, P., Matteoli, E. Thermochim. Acta **247**(2), 293–313 (1994) https://doi.org/10.1016/0040-6031(94)80129-0

[26] Conti, G., Gianni, P., Lepori, L., Matteoli, E., D'Amico, M.L. J. Chem. Thermodyn. **30**(7), 855–868 (1998) https://doi.org/10.1006/jcht.1997.0354

[27] Das, M., Roy, M.N. J. Chem. Eng. Data **51**(6), 2225–2232 (2006) https://doi.org/10.1021/je060311a

[28] Das, B., Roy, M.N., Hazra, D.K. Indian J. Chem. **1**(2), 93–97 (1994)

[29] Deshpande, D.D., Bhatgadde, L.G., Oswal, S., Prabhu, C.S. J. Chem. Eng. Data **16**(4), 469–473 (1971) https://doi.org/10.1021/je60051a007

[30] Dhaduk, B.B., Patel, C.B., Parsania, P.H. J. Sol. Chem. **44**(10), 1976–1996 (2015) https://doi.org/10.1007/s10953-015-0389-y

[31] Fattahi, M., Iloukhani, H. J. Chem. Thermodyn. **42**(11), 1335–1345 (2010) https://doi.org/10.1016/j.jct.2010.05.009

[32] Fenclová, D., Dohnal, V. J. Chem. Thermodyn. **23**(10), 911–921 (1991) https://doi.org/10.1016/S0021-9614(05)80172-7

[33] Francesconi, R., Lunelli, B., Comelli, F. Thermochim. Acta **207**, 45–52 (1992) https://doi.org/10.1016/0040-6031(92)80122-D

[34] Francesconi, R., Comelli, F. J. Chem. Eng. Data **39**(1), 106–107 (1994) https://doi.org/10.1021/je00013a028

[35] Francesconi, R., Comelli, F. J. Chem. Eng. Data **40**(1), 31–33 (1995) https://doi.org/10.1021/je00017a008

[36] Francesconi, R., Comelli, F. J. Chem. Eng. Data **40**(2), 512–514 (1995) https://doi.org/10.1021/je00018a035

[37] Francesconi, R., Comelli, F. J. Chem. Eng. Data **37**(2), 230–232 (1992) https://doi.org/10.1021/je00006a025

[38] Francesconi, R., Comelli, F., Malta, V. J. Chem. Eng. Data **38**(3), 424–427 (1993) https://doi.org/10.1021/je00011a025

[39] Francesconi, R., Comelli, F., Bigi, A., Rubini, K. Thermochim. Acta **447**(2), 154–160 (2006) https://doi.org/10.1016/j.tca.2006.05.010

[40] Gadžurić, S., Nikolić, A., Vraneš, M., Jović, B., Damjanović, M., Dožić, S. J. Chem. Thermodyn. **51**, 37–44 (2012) https://doi.org/10.1016/j.jct.2012.02.033

[41] Gascón, I., Lafuente, C., Cea, P., Royo, F.M., Urieta, J.S. Fluid Phase Equilib. **164**(1), 143–155 (1999) https://doi.org/10.1016/S0378-3812(99)00257-5

[42] Gascón, I., Lafuente, C., Cea, P., Domínguez, M., Royo, F.M. Int. J. Thermophys. **21**(5), 1185–1196 (2000) https://doi.org/10.1023/A:1026406323039

[43] Geppert-Rybczyńska, M., Sitarek, M. J. Chem. Eng. Data **59**(4), 1213–1224 (2014) https://doi.org/10.1021/je400781b

[44] Gill, D.S., Singh, J., Ludwig, R., Zeidler, M.D. Faraday Trans. **89**(21), 3955 (1993) https://doi.org/10.1039/FT9938903955

[45] Giner, B., Gascón, I., Villares, A., Cea, P., Lafuente, C. J. Chem. Eng. Data **51**(4), 1321–1325 (2006) https://doi.org/10.1021/je0600653

[46] Giner, B., Bandrés, I., López, M.C., Lafuente, C., Galindo, A. J. Chem. Phys. **127**(14), 144513 (2007) https://doi.org/10.1063/1.2773722

[47] Giner, B., Villares, A., Martín, S., Lafuente, C., Royo, F.M. Fluid Phase Equilib. **251**(1), 8–16 (2007) https://doi.org/10.1016/j.fluid.2006.10.024

[48] Govender, U.P., Letcher, T.M., Garg, S.K., Ahluwalia, J.C. J. Chem. Eng. Data **41**(1), 147–150 (1996) https://doi.org/10.1021/je9501494

[49] Grolier, J.P.E., Inglese, A., Wilhelm, E. J. Chem. Eng. Data **27**(3), 333–335 (1982) https://doi.org/10.1021/je00029a031

[50] Gupta, M., Vibhu, I., Shukla, J.P. Phys. Chem. Liq. **41**(6), 575–582 (2003) https://doi.org/10.1080/00319100310001613038

[51] Hnědkovský, L., Cibulka, I. J. Chem. Thermodyn. **18**(4), 331–337 (1986) https://doi.org/10.1016/0021-9614(86)90078-9

[52] Holland, R.S., Smyth, C.P. J. Phys. Chem. **59**(10), 1088–1092 (1955) https://doi.org/10.1021/j150532a025

[53] Ijardar, S.P., Malek, N.I. J. Chem. Thermodyn. **71**, 236–248 (2014) https://doi.org/10.1016/j.jct.2013.11.027

[54] Iloukhani, H., Zoorasna, N., Soleimani, R. Phys. Chem. Liq. **43**(4), 391–401 (2005) https://doi.org/10.1080/00319100500134048

[55] Inglese, A., Grolier, J.P.E., Wilhelm, E. J. Chem. Eng. Data **28**(1), 124–127 (1983) https://doi.org/10.1021/je00031a032

[56] Ivanov, E.V. J. Chem. Thermodyn. **43**(1), 58–62 (2011) https://doi.org/10.1016/j.jct.2010.08.006

[57] Ivanov, E.V. J. Chem. Thermodyn. **72**, 37–43 (2014) https://doi.org/10.1016/j.jct.2013.12.028

[58] Jagodzinski, P., Petrucci, S. J. Phys. Chem. **78**(9), 917–925 (1974) https://doi.org/10.1021/j100602a013

[59] Jain, D.V.S., Saini, S.B., Chaudhry, V. Indian J. Chem. **3**(18), 198–200 (1979)

[60] Jatkar, S., Deshpande, C. J. Ind. Chem. Soc. **37**, 1–10 (1960)

[61] Jha, A., Choudhury, A., Jha, M., Gurung, B.B., Mahendra, N.R. J. Ind. Chem. Soc. **80**(10), 886–893 (2003)

[62] Kalali, H.E., Demiriz, A.M., Budde, J., Kohler, F., Dallos, A., Ratkovics, F. Fluid Phase Equilib. **54**, 111–120 (1990) https://doi.org/10.1016/0378-3812(90)85074-K

[63] Katuno, M. Bull. Chem. Soc. Jap. **21**(7-12), 65–68 (1948) https://doi.org/10.1246/bcsj.21.65

[64] Kinart, C.M., Kinart, W.J., Ćwiklińska, A. J. Therm. Anal. Calorim: **68**(1), 307–317 (2002) https://doi.org/10.1023/A:1014981921097

[65] Kiyohara, O., D'Arcy, P.J., Benson, G.C. Can. J. Chem. **57**(9), 1006–1010 (1979)

https://doi.org/10.1139/v79-167

[66] Klages, F., Möhler, K. Chem. Ber. **81**(5), 411–417 (1948) https://doi.org/10.1002/cber.19480810512

[67] Knežević-Stevanović, A.B., Šerbanović, S.P., Radović, I.R., Djordjević, B.D., Kijevčanin, M.L. J. Chem. Eng. Data **58**(11), 2932–2951 (2013) https://doi.org/10.1021/je4003916

[68] Knežević-Stevanović, A.B., Smiljanic, J., Serbanovic, S., Radović, I.R., Kijevcanin, M. J. Serb. Chem. Soc. **79**(1), 77–87 (2014) https://doi.org/10.2298/JSC130407045K

[69] Komatsu, S., Masumoto, M. Bull. Chem. Soc. Jap. **5**(8), 241–248 (1930) https://doi.org/10.1246/bcsj.5.241

[70] Korösi, G., Kovats, E.S. J. Chem. Eng. Data **26**(3), 323–332 (1981) https://doi.org/10.1021/je00025a032

[71] Ku, H.-C., Wang, C.-C., Tu, C.-H. J. Chem. Eng. Data **53**(2), 566–573 (2008) https://doi.org/10.1021/je700626v

[72] Kumar, A. J. Chem. Eng. Data **45**(4), 630–635 (2000) https://doi.org/10.1021/je990261s

[73] Kushare, S.K., Kolhapurkar, R.R., Dagade, D.H., Patil, K.J. J. Chem. Eng. Data **51**(5), 1617–1623 (2006) https://doi.org/10.1021/je0601098

[74] Lafuente, C., Cea, P., Domínguez, M., Royo, F.M., Urieta, J.S. J. Sol. Chem. **30**(9), 795–805 (2001) https://doi.org/10.1023/A:1012284115975

[75] Lejček, P., Matouš, J., Novák, J.P., Pick, J. J. Chem. Thermodyn. **7**(10), 927–935 (1975) https://doi.org/10.1016/0021-9614(75)90156-1

[76] Lepori, L., Matteoli, E. J. Chem. Thermodyn. **18**(1), 13–19 (1986) https://doi.org/10.1016/0021-9614(86)90037-6

[77] Letcher, T.M., Domańska, U. J. Chem. Thermodyn. **26**(2), 113–119 (1994) https://doi.org/10.1006/jcht.1994.1027

[78] Letcher, T.M., Domańska, U. J. Chem. Thermodyn. **26**(11), 1241–1247 (1994) https://doi.org/10.1006/jcht.1994.1141

[79] Loras, S., Aucejo, A., Muñoz, R., Wisniak, J. J. Chem. Eng. Data **44**(3), 583–587 (1999) https://doi.org/10.1021/je980258t

[80] Loras, S., Aucejo, A., Montón, J.B., Wisniak, J., Segura, H. J. Chem. Eng. Data **47**(5), 1256–1262 (2002) https://doi.org/10.1021/je0255325

[81] Marczak, W., Sajewicz, M., Bucek, M., Piotrowski, D., Szewczyk, K., Kowalska, T. J. Mol. Liq. **141**(1-2), 8–16 (2008) https://doi.org/10.1016/j.molliq.2008.02.004

[82] Mariano, A., Camacho, A., Postigo, M., Valen, A., Artigas, H., Royo, F.M., Urieta, J.S. Braz. J. Chem. Eng. **17**, 459–470 (2000) https://doi.org/10.1590/S0104-66322000000400011

[83] Matouš, J., Novák, J.P., Šobr, J., Pick, J. Collect. Czech. Chem. Commun. **37**(8), 2653–2663 (1972) https://doi.org/10.1135/cccc19722653

[84] Matsuda, H., Kamihama, N., Kurihara, K., Tochigi, K., Yokoyama, K. J. Chem. Eng. Japan **44**(3), 131–139 (2011) https://doi.org/10.1252/jcej.10we162

[85] Metz, D.J., Glines, A. J. Phys. Chem. **71**(4), 1158 (1967) https://doi.org/10.1021/j100863a067

[86] Muhuri, P.K., Das, B., Hazra, D.K. J. Chem. Eng. Data **41**(6), 1473–1476 (1996) https://doi.org/10.1021/je960196b

[87] Nain, A.K. J. Sol. Chem. **35**(10), 1417–1439 (2006) https://doi.org/10.1007/s10953-006-9071-8

[88] Nain, A.K., Droliya, P. J. Chem. Thermodyn. **105**, 317–326 (2017) https://doi.org/10.1016/j.jct.2016.10.042

[89] Naorem, H., Suri, S.K. Can. J. Chem. **67**(10), 1672–1675 (1989) https://doi.org/10.1139/v89-256

[90] Nayak, J.N., Aralaguppi, M.I., Toti, U.S., Aminabhavi, T.M. J. Chem. Eng. Data **48**(6), 1483–1488 (2003) https://doi.org/10.1021/je030147g

[91] Nayak, J.N., Aralaguppi, M.I., Kumar Naidu, B.V., Aminabhavi, T.M. J. Chem. Eng. Data **49**(3), 468–474 (2004) https://doi.org/10.1021/je030196t

[92] Nicolas, M., Malineau, M., Reich, R. Phys. Chem. Liq. **10**(1), 11–22 (1980) https://doi.org/10.1080/00319108008078453

[93] Nikolić, A., Gobor, L., Krstić, V., Petrović, S. J. Mol. Liq. **121**(2-3), 139–142 (2005) https://doi.org/10.1016/j.molliq.2004.12.001

[94] Nikolić, A., Gobor, L., Despotović, V., Majkić, S. J. Mol. Liq. **126**(1-3), 95–98 (2006) https://doi.org/10.1016/j.molliq.2005.09.008

[95] Nonay, N., Giner, I., Giner, B., Artigas, H., Lafuente, C. Fluid Phase Equilib. **295**(1), 130–136 (2010) https://doi.org/10.1016/j.fluid.2010.04.017

[96] Oshmyansky, Y., Hanley, H.J.M., Ely, J.F., Kidnay, A.J. Int. J. Thermophys.

7(3), 599–608 (1986) https://doi.org/10.1007/BF00502393

[97] Oswal, S.L., Gardas, R.L., Phalak, R.P. Thermochim. Acta **426**(1-2), 199–206 (2005) https://doi.org/10.1016/j.tca.2004.08.001

[98] Oswal, S.L., Gardas, R.L., Phalak, R.P. J. Mol. Liq. **116**(2), 109–118 (2005) https://doi.org/10.1016/j.molliq.2004.07.081

[99] Oswal, S.L., Pandiyan, V., Krishnakumar, B., Vasantharani, P. Thermochim. Acta **507-508**, 27–34 (2010) https://doi.org/10.1016/j.tca.2010.04.025

[100] Ottani, S., Vitalini, D., Comelli, F., Castellari, C. J. Chem. Eng. Data **47**(5), 1197–1204 (2002) https://doi.org/10.1021/je020030c

[101] Ottani, S., Francesconi, R., Comelli, F., Castellari, C. Thermochim. Acta **401**(2), 87–93 (2003) https://doi.org/10.1016/S0040-6031(02)00501-4

[102] Palani, R., Geetha, A. Phys. Chem. Liq. **47**(5), 542–552 (2009) https://doi.org/10.1080/00319100802562862

[103] Palani, R., Saravanan, S., Kumar, R.: **2**(3), 622–629 (2009)

[104] Pandiyan, V., Oswal, S.L., Malek, N.I., Vasantharani, P. Thermochim. Acta **524**(1-2), 140–150 (2011) https://doi.org/10.1016/j.tca.2011.07.005

[105] Peralta, R.D., Infante, R., Cortis, G., Ramos, L.F., Wisniak, J. Phys. Chem. Liq. **41**(4), 361–369 (2003) https://doi.org/10.1080/0031910031000122052

[106] Peralta, R.D., Infante, R., Cortez, G., Wisniak, J. Phys. Chem. Liq. **43**(3), 249–259 (2005) https://doi.org/10.1080/00319100500061274

[107] Pérez, E., Cardoso, M., Mainar, A.M., Pardo, J.I., Urieta, J.S. J. Chem. Eng. Data **48**(5), 1306–1309 (2003) https://doi.org/10.1021/je034076x

[108] Piñeiro, Á., Brocos, P., Amigo, A., Pintos, M., Bravo, R. J. Sol. Chem. **31**(5), 369–380 (2002) https://doi.org/10.1023/A:1015807331250

[109] Postigo, M., Mariano, A., Mussari, L., Camacho, A., Urieta, J. Fluid Phase Equilib. **207**(1-2), 193–207 (2003) https://doi.org/10.1016/S0378-3812(03)00021-9

[110] Prasad, T.E.V., Raju, A.N., Sriram, N., Prasad, D.H.L. Fluid Phase Equilib. **227**(1), 37–39 (2005) https://doi.org/10.1016/j.fluid.2004.10.027

[111] Qun-Fang, L., Rui-Sen, L., Dan-Yan, N., Yu-Chun, H. J. Chem. Eng. Data **42**(5), 971–974 (1997) https://doi.org/10.1021/je960351m

[112] Ramkumar, D.H.S., Kudchadker, A.P. J. Chem. Eng. Data **34**(4), 463–465 (1989) https://doi.org/10.1021/je00058a027

[113] Rathnam, M.V. J. Chem. Eng. Data **33**(1), 14–15 (1988) https://doi.org/10.1021/je00051a006

[114] Rathnam, M.V., Ambavadekar, D.R., Nandini, M. J. Chem. Eng. Data **58**(12), 3370–3377 (2013) https://doi.org/10.1021/je400539h

[115] Rathnam, M.V., Ambavadekar, D.R., Nandini, M. J. Mol. Liq. **187**, 58–65 (2013) https://doi.org/10.1016/j.molliq.2013.06.002

[116] Rodnikova, M.N., Gunina, M.A., Makarov, D.M., Egorov, G.I., Val'kovskaya, T.M. Russ. J. Phys. Chem. **85**(9), 1676–1678 (2011) https://doi.org/10.1134/S003602441109024X

[117] Rodríguez, S., Lafuente, C., Carrin, J.A., Royo, F.M., Urieta, J.S. Int. J. Thermophys. **17**(6), 1281–1288 (1996) https://doi.org/10.1007/BF01438670

[118] Rodríguez, S., Lafuente, C., Cea, P., Royo, F.M., Urieta, J.S. J. Chem. Eng. Data **42**(6), 1285–1289 (1997) https://doi.org/10.1021/je970149z

[119] Rodríguez, S., Lafuente, C., Artigas, H., Royo, F.M., Urieta, J.S. J. Chem. Thermodyn. **31**(1), 139–149 (1999) https://doi.org/10.1006/jcht.1998.0437

[120] Rodríguez, S., Artigas, H., Lafuente, C., Mainar, A.M., Royo, F.M. Thermochim. Acta **362**(1-2), 153–160 (2000) https://doi.org/10.1016/S0040-6031(00)00580-3

[121] Rodríguez, S., Giner, B., Haro, M., Martín, S., Artigas, H. Phys. Chem. Liq. **44**(3), 275–285 (2006) https://doi.org/10.1080/00319100600574168

[122] Roy, M.N., Dey, R., Jha, A. J. Chem. Eng. Data **46**(5), 1327–1329 (2001) https://doi.org/10.1021/je010009w

[123] Saleh, M.A., Akhtar, S., Ahmed, M.S., Uddin, M.H. Phys. Chem. Liq. **40**(5), 621–635 (2002) https://doi.org/10.1080/0031910029001/0473

[124] Schedemann, A.: Aufbau und Inbetriebnahme einer Dichtemessanlage. Messung und Modellierung des PVT-Verhaltens bis zu Drücken von 1400 bar. Diplomarbeit, Universität Oldenburg, Oldenburg (April 2009)

[125] Schornack, L.G., Eckert, C.A. J. Phys. Chem. **74**(15), 3014–3020 (1970) https://doi.org/10.1021/j100709a030

[126] Segura, H., Mejía, A., Reich, R., Wisniak, J., Loras, S. Phys. Chem. Liq. **41**(3), 283–301 (2003) https://doi.org/10.1080/0031910021000044456

[127] Shelar, R.N., Patil, A.V., Dighavkar, C.G., Borse, R.Y. Indian J. Pure Appl. Phys. **54**(7), 463–470 (2016) https://doi.org/10.56042/ijpap.v54i7.4627

[128] Sinha, A., Roy, M.N. Phys. Chem. Liq. **44**(3), 303–314 (2006) https://doi.org/

10.1080/00319100600576809

[129] Sinha, A., Roy, M.N. J. Chem. Eng. Data **51**(4), 1415–1423 (2006) https://doi.org/10.1021/je060113j

[130] Sinha, B., Pradhan, R., Saha, S., Brahman, D., Sarkar, A. J. Serb. Chem. Soc. **78**(9), 1443–1460 (2013) https://doi.org/10.2298/JSC121210031S

[131] Smyth, C.P., Walls, W.S. J. Am. Chem. Soc. **54**(8), 3230–3240 (1932) https://doi.org/10.1021/ja01347a027

[132] Solimo, H.N., Gomez Marigliano, A.C. J. Sol. Chem. **22**(10), 951–962 (1993) https://doi.org/10.1007/BF00646606

[133] Surendranath, K.N., Chandrasekhar, A.C.H., Krishnaiah, A. Phys. Chem. Liq. **24**(4), 255–259 (1992) https://doi.org/10.1080/00319109208027277

[134] Suri, S.K., Naorem, H. J. Chem. Eng. Data **32**(4), 462–464 (1987) https://doi.org/10.1021/je00050a023

[135] Taniewska-Osińska, S., Nowicka, B., Kacperska, A., Bald, A. Phys. Chem. Liq. **25**(2), 113–125 (1993) https://doi.org/10.1080/00319109308030352

[136] Tôrres, R.B., Ortolan, M.I., Volpe, P.L.O. J. Chem. Thermodyn. **40**(3), 442–459 (2008) https://doi.org/10.1016/j.jct.2007.09.007

[137] Vaid, Z., More, U., Ijardar, S.P., Malek, N.I. J. Chem. Thermodyn. (86), 143–153 (2015) https://doi.org/10.1016/j.jct.2015.02.011

[138] Valén, A., López, M.C., Urieta, J.S., Royo, F.M., Lafuente, C. J. Mol. Liq. **95**(2), 157–165 (2002) https://doi.org/10.1016/S0167-7322(01)00279-3

[139] Valén, A., Gascón, I., Lafuente, C., Urieta, J.S., Royo, F.M., Postigo, M. Int. J. Thermophys. **23**(6), 1587–1598 (2002) https://doi.org/10.1023/A:1020892201382

[140] Valén, A., Gascón, I., Lafuente, C., López, M.C., Royo, F.M. Phys. Chem. Liq. **41**(3), 239–247 (2003) https://doi.org/10.1080/0031910031000079907

[141] Vercher, E., Orchillés, A.V., Llopis, F.J., González-Alfaro, V., Martínez-Andreu, A. J. Chem. Eng. Data **56**(12), 4633–4642 (2011) https://doi.org/10.1021/je200609g

[142] Villares, A., Rodríguez, S., Lafuente, C., Royo, F.M., López, M.C. J. Sol. Chem. **33**(9), 1119–1133 (2004) https://doi.org/10.1023/B:JOSL.0000048060.42529.6a

[143] Vittal Prasad, T.E., Raj, E.D.A., Maheedhar, G., Reddy, M.S., Kumar, V.S., Garapati, S., Patanjali, V., Prasad, D.H.L. J. Chem. Eng. Data **49**(4), 746–749

(2004) https://doi.org/10.1021/je030141r

[144] Wang, J., Zhao, Y., Zhuo, K., Lin, R. Can. J. Chem. **80**(7), 753–760 (2002) https://doi.org/10.1139/v02-092

[145] Wang, Z., Benson, G.C., Lu, B.C.-Y. J. Chem. Thermodyn. **35**(10), 1635–1644 (2003) https://doi.org/10.1016/S0021-9614(03)00148-4

[146] Wankhede, D.S., Wankhede, N.N., Lande, M.K., Arbad, B.R. J. Mol. Liq. **138**(1-3), 124–129 (2008) https://doi.org/10.1016/j.molliq.2007.07.008

[147] Wankhede, D.S., Wankhede, N.N., Arbad, B.R., Lande, M.K. Int. J. Thermophys. **31**(11-12), 2239–2245 (2010) https://doi.org/10.1007/s10765-010-0860-3

[148] Weissler, A. J. Am. Chem. Soc. **71**(2), 419–421 (1949) https://doi.org/10.1021/ja01170a014

[149] Whitehead, E.V., Dean, R.A., Fidler, F.A. J. Am. Chem. Soc. **73**(8), 3632–3635 (1951) https://doi.org/10.1021/ja01152a022

[150] Zafarani-Moattar, M.T., Majdan-Cegincara, R. J. Chem. Eng. Data **52**(6), 2359–2364 (2007) https://doi.org/10.1021/je700338t

[151] Zikmundová, D., Matouš, J., Novák, J.P., Kubíček, V., Pick, J. Fluid Phase Equilib. **54**, 93–110 (1990) https://doi.org/10.1016/0378-3812(90)85073-J

[152] Živković, E.M., Bajić, D.M., Radović, I.R., Šerbanović, S.P., Kijevčanin, M.L. Fluid Phase Equilib. **373**, 1–19 (2014) https://doi.org/10.1016/j.fluid.2014.04.002

[153] Zurita, J.L., Garcia, D.A., Postigo, M.A. J. Chem. Eng. Data **37**(2), 206–209 (1992) https://doi.org/10.1021/je00006a018
